# Ocular manifestations of congenital anomalies of the kidney and urinary tract (CAKUT)

**DOI:** 10.1007/s00467-023-06068-9

**Published:** 2023-07-20

**Authors:** James Virth, Heather G. Mack, Deb Colville, Emma Crockett, Judy Savige

**Affiliations:** 1 Department of Medicine (Melbourne Health and Northern Health), Royal Melbourne Hospital, The University of Melbourne, Parkville, VIC 3050 Australia; 2 University Department of Surgery (Ophthalmology), Royal Victorian Eye and Ear Hospital, East Melbourne, VIC 3002 Australia

**Keywords:** Congenital anomalies of the kidney and urinary tract (CAKUT), Coloboma, Microphthalmia, Optic disc anomalies, Refraction errors, Cataract

## Abstract

Congenital anomalies of the kidney and urinary tract (CAKUT) are among the most common birth defects worldwide and a major cause of kidney failure in children. Extra-renal manifestations are also common. This study reviewed diseases associated with the Genomics England CAKUT-associated gene panel for ocular anomalies. In addition, each gene was examined for expression in the human retina and an ocular phenotype in mouse models using the Human Protein Atlas and Mouse Genome Informatics databases, respectively. Thirty-four (54%) of the 63 CAKUT-associated genes (55 ‘green’ and 8 ‘amber’) had a reported ocular phenotype. Five of the 6 most common CAKUT-associated genes (*PAX2*, *EYA1*, *SALL1*, *GATA3*, *PBX1)* that represent 30% of all diagnoses had ocular features. The ocular abnormalities found with most CAKUT-associated genes and with five of the six commonest were coloboma, microphthalmia, optic disc anomalies, refraction errors (astigmatism, myopia, and hypermetropia), and cataract. Seven of the CAKUT-associated genes studied (11%) had no reported ocular features but were expressed in the human retina or had an ocular phenotype in a mouse model, which suggested further possibly-unrecognised abnormalities. About one third of CAKUT-associated genes (18, 29%) had no ocular associations and were not expressed in the retina, and the corresponding mouse models had no ocular phenotype. Ocular abnormalities in individuals with CAKUT suggest a genetic basis for the disease and sometimes indicate the affected gene. Individuals with CAKUT often have ocular abnormalities and may require an ophthalmic review, monitoring, and treatment to preserve vision.

**Supplementary Information:**

The online version contains supplementary material available at 10.1007/s00467-023-06068-9.

## CAKUT

Genetic kidney disease comprises congenital anomalies of the kidney and urinary tract (CAKUT), cystic kidney diseases and ciliopathies, glomerulopathies such as Alport syndrome, complementopathies, and focal and segmental glomerulosclerosis (FSGS), as well as the tubulopathies.

CAKUT are a diverse group of developmental anomalies and the most common (20–30%) of all birth defects [1]. CAKUT is found in nearly 1% of live births worldwide, and is the cause in almost half the children who develop kidney failure [1].

The kidney phenotypes of CAKUT include agenesis, hypoplasia, dysplasia, cysts, ectopia, fusion, hydronephrosis, urinary tract agenesis, duplication, megaureter, ureteropelvic junction obstruction, vesicoureteral reflux, and posterior urethral valves. Defects may be unilateral or bilateral. Some changes, such as bilateral kidney agenesis, are almost always fatal [2]. Some progress to kidney failure necessitating dialysis or transplantation within the first few years of life or later [3]. Some have an excellent prognosis [3]. At present, many individuals with CAKUT may be diagnosed antenatally with ultrasound but others are found incidentally after imaging, repeated urinary tract infections, or the detection of impaired kidney function.

CAKUT occurs in isolation or together with defects in other organ systems in syndromic disease. Many cases are sporadic [4], with no identifiable cause, and probably result from both genetic and environmental factors, including maternal diabetes or obesity [5]. However, familial CAKUT consistent with a genetic basis still represents at least 10–20% of cases [4].

More than 150 genes are associated with CAKUT and more remain to be identified. Many of these genes are transcription factors that are important in embryonic development. Six are common (*PAX2*, *HNF1B*, *EYA1*, *SALL1*, *GATA3*, *PBX1*) but many of the others are found only in individual families [6]. Inheritance is usually autosomal dominant (AD) with incomplete penetrance and variable expression. This means that clinical features vary even within a family, and one affected family member may have a single kidney, another has vesicoureteric reflux and another affected family member has normal kidneys. CAKUT may be misdiagnosed as FSGS since some genes result in a reduced nephron number. In addition, the diagnosis of CAKUT may be overlooked when it results from copy number variants that are difficult to detect with Whole Exome Sequencing.

Ocular anomalies occur in different forms of CAKUT. This is because of the developmental and structural overlap between the kidney and the eye despite their different functions. The kidney and the eye develop embryologically at about the same time (5^th^ to 12^th^ weeks of gestation) and under the control of some of the same transcription factors, including *BMP7*, *EYA1*, *FOXC1*, *PAX*, and *WNT1* [7]. Surprisingly, the kidney and the eye also share structural features such as the epithelial cell barrier, basement membrane and capillary network in the glomerular filter and in the retinal pigment epithelial cells, Bruch’s membrane and choriocapillaris [7]. In addition, the glomerular and retinal basement membranes both mainly comprise the collagen IV α3α4α5 network. Importantly the kidney and the retina also depend on ciliated cells for their functions.

This review examines monogenic causes of CAKUT for their ocular associations. Identifying ocular features suggests that the disease has a genetic basis, and may indicate the affected gene and the need for treatment or ophthalmic monitoring to prevent complications.

## Search strategy for ocular features in CAKUT

Sixty-three genes associated with CAKUT from the ‘green’ (*n* = 55) or ‘amber’ lists (*n* = 8) of the Genomics England CAKUT panel (https://panelapp.genomicsengland.co.uk/) were searched for ocular manifestations in the Online Mendelian Inheritance in Man (OMIM, https://www.omim.org/), PubMed and Google Scholar (https://scholar.google.com/) databases between July 2022 and March 2023. Genomics England uses a traffic light system where green genes have a high level of evidence for a disease association (reported in 3 unrelated families or 2 families with additional strong evidence) as decided by an expert panel. These panels represent the genes often examined in a diagnostic genetic laboratory. Amber and red genes have borderline and low levels of evidence for disease association.

The 63 genes were also examined for mRNA expression in the retina in The Human Protein Atlas (https://www.proteinatlas.org), and for an ocular phenotype in mouse models in the Mouse Genome Informatics (MGI) database (http://www.informatics.jax.org/).

## Ocular features associated with CAKUT

About half of the 63 CAKUT-associated genes in the Genomics England panels (34, 54%) have reported ocular abnormalities (Supplementary Table 1). About one third of CAKUT genes (18, 29%) have no reported ocular associations, are not expressed in the human retina and have no ocular features in a mouse model. Seven further genes (7, 11%) have no reported ocular phenotype in human disease but are expressed in the human retina or the mouse models have ocular features. Thus, further ocular abnormalities may yet be described with these other genes.

Five of the 6 most common CAKUT genes that represent 30% of all cases (*PAX2*, *EYA1*, *SALL1*, *GATA3*, *PBX1)* have an ocular phenotype (Table 1). There are no reported ocular features for the *HNF1B* gene which is affected in HNF1B-nephropathy, formerly known as renal cysts and diabetes syndrome.

**Table 1:** Most common CAKUT genes, their ocular associations, retinal expression, and ocular phenotypes in mouse models

Gene	Disease, OMIM number	Renal features	Extrarenal features (OMIM)	Ocular features	mRNA expression (Human Protein Atlas)	Knockout mouse model (MGI)	Reference
*HNF1B (189907)*	Renal cysts and diabetes syndrome (AD);137920	Renal agenesis, hypoplasia, dysplasia, cysts, horseshoe kidney; ureteropelvic junction obstruction	Gout, diabetes, hypomagnesemia	None reported	NA	None noted	Bellanne-Chantelot et al. (2004) [8]; Kaplan et al. (1989) [9]; Nakayama et al. (2010) [10]; Rizzoni et al. (1982) [11]
*PAX2 (167409)*	Papillorenal syndrome (renal-coloboma syndrome) (AD); 120330	Kidney agenesis, hypoplasia, dysplasia, cysts, malrotation, horseshoe kidney; pyeloureteric duplication; hydro-nephrosis, ureteropelvic junction obstruction, ureterovesical junction obstruction, vesicoureteric reflux	Hearing loss, joint laxity	Reduced visual acuity, visual field defects; microphthalmia; coloboma (retina, optic nerve); microcornea; lens opacity, posterior lens subluxation; retinal staphyloma, retinal hypoplasia, abnormal retinal pigment epithelium, macular hyperpigmentation, chorioretinal degeneration, cystic macular degeneration, retinal detachment; optic disc dysplasia, ‘morning glory’ anomaly, optic pit, rudimentary/ absent central retinal vessels, optic disc hypoplasia, aplasia, hyperplasia	1.2 TPM	Abnormal retinal vasculature, abnormal optic nerve, optic disc coloboma, abnormal retinal pigmentation, abnormal retinal nerve fibre layer	Eccles and Schimmenti (1999) [12]; Schimmenti (2011) [13]
*EYA1 (601653)*	Branchiootorenal syndrome 1, with or without cataract(AD) or Anterior segment anomalies with or without cataract; 113650	Kidney agenesis, hypoplasia, dysplasia, cysts, crossed ectopia, malrotation; distorted pelvicalyceal system, bifid kidney pelvis, narrowed ureteropelvic junction; ureteral duplication; vesicoureteric reflux	Hearing loss, preauricular pits, abnormal ears, cochlear malformation, high arched palate	Congenital nuclear-type cataracts; nystagmus, esotropia; reduced visual acuity; central corneal opacity (Peters’ anomaly); abnormality of the lacrimal ducts	0.4 TPM	Eyelids open at birth	Azuma et al. (2000) [14]; Carmi et al. (1983) [15]; Chitayat et al. (1992) [16]; Fraser et al. (1978) [17]; Fraser et al. (1983) [18]; Legius et al. (1990) [19]; Melnick et al. (1975) [20]; Melnick et al. (1976) [21]
*SALL1 (602218)*	Townes-Brocks branchio-oto-renal-like syndrome (AD);107480	Kidney agenesis, hypoplasia, dysplasia, cysts, ectopia (pelvic kidney); vesicoureteric reflux; urethral valves, stenosis	Skeletal, facial, cardiac, gastrointestinal anomalies; hearing loss	Unilateral microphthalmia; coloboma (iris, choroid, retina); congenital lamellar cataract; optic nerve atrophy; Duane anomaly (limited horizontal eye movement); orbital dermoid; crocodile tears	4.6 TPM	None noted	Blanck et al. (2000) [22]; Botzenhart et al. (2005) [23]; Botzenhart et al. (2007) [24]; Ferraz et al. (1989) [25]; Johnson et al. (1996) [26]; Kohlhase et al. (1999) [27]; Kurnit et al. (1978) [28]; Rossmiller and Pasic (1994) [29]
*GATA3 (131320)*	Hypoparathyroidism, sensorineural deafness, and renal dysplasia (AD); 146255	Kidney agenesis, hypoplasia, dysplasia; vesicoureteric reflux	Hearing loss, abnormal female genitalia	Inherited retinal degeneration; pseudopapilloedema; horizontal nystagmus	NA	Narrow eye opening	Barakat et al. (2018) [30]; Bilous et al. (1992) [31]; Ferraris et al. (2009) [32]; Hasegawa et al. (1997) [33]
*PBX1 (176310)*	CAKUT with or without hearing loss, abnormal ears, or developmental delay (AD); 617641	Kidney hypoplasia, dysplasia, cysts, ectopia, horseshoe kidney; dilated kidney/ calyces/pelvis, dilated ureter; bifid ureter; vesicoureteric reflux; urethral valve	Hearing loss; facial anomalies; ear anomalies including malformed pinnae; short stature; cardiac anomalies, abnormal genitalia developmental delay	Strabismus; corneal dystrophy and clouding present from birth; glaucoma; iris abnormalities	17.1 TPM	Eye lids open at birth	Heidet et al. (2017) [34]; Le Tanno et al. (2017) [35]; Slavotinek et al. (2017) [36]; Murphy et al. (2010) [37]; Safgren et al. (2021) [38]

TPM, transcripts per million; NA not available

**Fig. 1:**
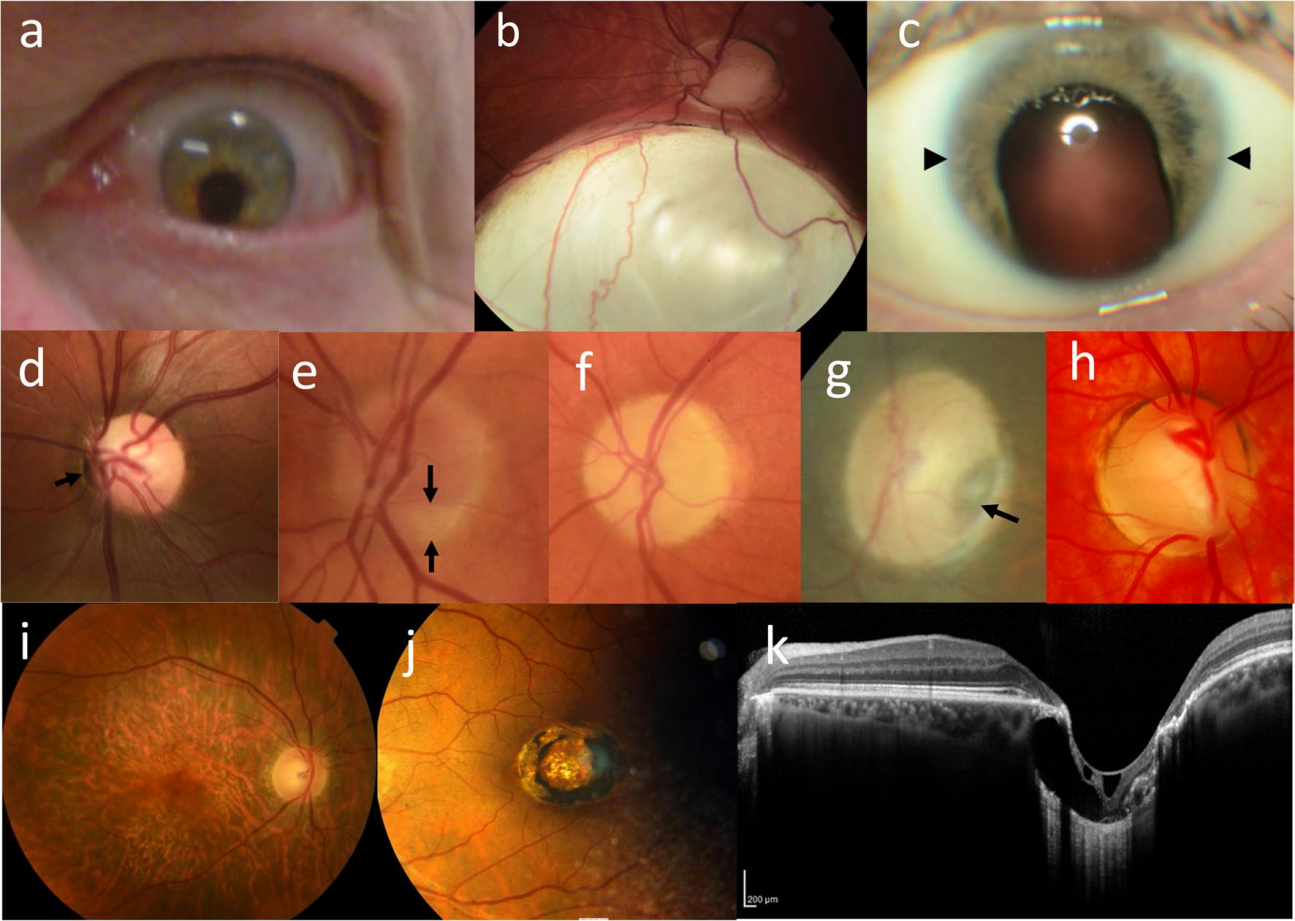
Common ocular abnormalities associated with CAKUT: **a** iris coloboma that resembles a ‘keyhole’ inferiorly; **b** large chorioretinal coloboma; **c** inferior iris coloboma with microphthalmia (diameter between arrows 7 mm); **d** subtle papilloretinal syndrome with vessels emerging from the periphery of the disc (arrow); **e** optic disc hypoplasia, arrows indicate area of missing disc; **f** optic atrophy; **g** optic disc pit; **h** optic disc coloboma; **i** inherited retinal dystrophy; **j** macular coloboma with excavated lesion and surrounding pigmented margin; and **k** optical coherence tomography scan of the fundus in **j** confirming macular coloboma with excavation, absent retina and choroid, and thinned residual sclera, but more distant normal retina and disc.

The most common ocular abnormalities associated with CAKUT-associated genes are coloboma, optic disc (‘morning glory’) anomalies, microphthalmia, refraction errors (astigmatism, myopia, and hypermetropia), and cataracts (Table 2, Fig. 1).

**Table 2:** Common ocular abnormalities associated with CAKUT-causing genes

Ophthalmic Finding	Genes and Syndromes
**Refractive error**	
Astigmatism	***CENPF*** (Stromme syndrome)
Hypermetropia	***CHD7*** (CHARGE syndrome); ***DDX6*** (Intellectual developmental disorder with impaired language and dysmorphic facies)
Myopia	***BMP4*** (Microphthalmia, syndromic 6); ***CHD7*** (CHARGE syndrome); ***JAG1*** (Alagille syndrome 1); ***KMT2D*** (Kabuki syndrome 1); ***NIPBL*** (Cornelia de Lange syndrome 1); ***PAX2*** (Papillorenal syndrome)
**Anomalies of globe size**	
Microphthalmia/anophthalmia	***BMP4*** (Microphthalmia, syndromic 6); ***CHD7*** (CHARGE syndrome); ***DHCR7*** (Smith-Lemli-Opitz syndrome); ***EP300*** (Rubinstein-Taybi syndrome 2); ***FRAS1*** (Fraser syndrome 1); ***FREM1*** (Manitoba oculotrichoanal syndrome); ***GLI3*** (Pallister-Hall syndrome); ***PAX2*** (Papillorenal syndrome); ***SALL1*** (Townes-Brocks syndrome 1; Townes-Brocks branchiootorenal-like syndrome); ***STRA6*** (Microphthalmia, syndromic 9; Microphthalmia, isolated, with coloboma 8); ***TFAP2A*** (Branchiooculofacial syndrome)
**Coloboma**	
Iris	***BMP4*** (Microphthalmia, syndromic 6); ***CENPF*** (Stromme syndrome); ***CHD7*** (CHARGE syndrome); ***SALL1*** (Townes-Brocks syndrome 1; Townes-Brocks branchiootorenal-like syndrome); ***STRA6*** (Microphthalmia, syndromic 9; Microphthalmia, isolated, with coloboma 8); ***TFAP2A*** (branchiooculofacial syndrome)
Retina and choroid	***BMP4*** (Microphthalmia, syndromic 6); ***CHD7*** (CHARGE syndrome); ***KMT2D*** (Kabuki syndrome 1); ***KRAS*** (Schimmelpenning-Feuerstein-Mims syndrome); ***PAX2*** (Papillorenal syndrome); ***SALL1*** (Townes-Brocks syndrome 1; Townes-Brocks branchiootorenal-like syndrome); ***SALL4*** (Duane-radial ray syndrome); ***STRA6*** (Microphthalmia, syndromic 9; Microphthalmia, isolated, with coloboma 8); ***TFAP2A*** (branchiooculofacial syndrome)
Optic nerve	***CHD7*** (CHARGE syndrome); ***PAX2*** (Papillorenal syndrome); ***SALL4*** (Duane-radial ray syndrome); ***TFAP2A*** (Branchiooculofacial syndrome)
**Anterior segment**	
Cataract	***CENPF*** (Stromme syndrome); ***CHD7*** (CHARGE syndrome); ***DHCR7*** (Smith-Lemli-Opitz syndrome); ***EYA1*** (Branchiootorenal syndrome 1, with or without cataracts; Anterior segment anomalies with or without cataract); ***GPC3*** (Simpson-Golabi-Behmel syndrome, type 1); ***JAG1*** (Alagille syndrome 1; ***LRP4*** (Cenani-Lenz syndactyly syndrome); ***PAX2*** (Papillorenal syndrome); ***SALL1*** (Townes-Brocks syndrome 1; Townes-Brocks Branchiootorenal-like syndrome); ***SALL4*** (Duane-radial ray syndrome); ***TFAP2A*** (Branchiooculofacial syndrome)
Ectopia lentis (dislocated lens)	***CHD7*** (CHARGE syndrome); ***PAX2*** (Papillorenal syndrome)
Iris hypoplasia	***CENPF*** (Stromme syndrome)
Iris cyst	***PLVAP*** (Diarrhea 10, protein-losing enteropathy type); ***TFAP2A*** (Branchiooculofacial syndrome)
Microcornea	***CENPF*** (Stromme syndrome); ***CHD7*** (CHARGE syndrome); ***JAG1*** (Alagille syndrome 1); ***SALL4*** (Duane-radial ray syndrome)
Corneal opacity	***CENPF*** (Stromme syndrome); ***EYA1*** (Anterior segment anomalies with or without cataract); ***JAG1*** (Alagille syndrome 1)
Peters’ anomaly (thinning and clouding of the cornea, and attachment to iris)	***CENPF*** (Stromme syndrome); ***EYA1*** (Anterior segment anomalies with or without cataract); ***NIPBL*** (Cornelia de Lange syndrome 1)
Sclerocornea	***CENPF*** (Stromme syndrome); ***STRA6*** (Microphthalmia, syndromic 9; Microphthalmia, isolated, with coloboma 8)
Corectopia (off-centre pupil)	***CENPF*** (Stromme syndrome); ***JAG1*** (Alagille syndrome 1)
Polycoria (two pupils)	***TFAP2A*** (Branchiooculofacial syndrome)
Posterior embryotoxon (peripheral corneal stroma)	***JAG1*** (Alagille syndrome 1); ***NOTCH2*** (Alagille syndrome 2)
Axenfeld-Rieger anomaly (posterior embryotoxon plus congenital iris anomalies)	***JAG1*** (Alagille syndrome 1)
**Posterior segment**	
Inherited retinal degeneration	***BMP4*** (Microphthalmia, syndromic 6); ***GATA3*** (Hypoparathyroidism, sensorineural deafness, and renal dysplasia) ***PAX2*** (Papillorenal syndrome)
Chorioretinal scar	***SALL4*** (Duane-radial ray syndrome)
Choroidal folds	***JAG1*** (Alagille syndrome 1)
Glaucoma	***DHCR7*** (Smith-Lemli-Opitz syndrome)
Optic atrophy	***DHCR7*** (Smith-Lemli-Opitz syndrome); ***SALL1*** (Townes-Brocks syndrome 1; Townes-Brocks branchiootorenal-like syndrome)
Optic nerve aplasia, dysplasia, hypoplasia	***PAX2*** (Papillorenal syndrome); ***STRA6*** (Microphthalmia, syndromic 9; Microphthalmia, isolated, with coloboma 8); ***SALL4*** (Duane-radial ray syndrome); ***CENPF*** (Stromme syndrome); ***DHCR7*** (Smith-Lemli-Opitz syndrome);
Optic disc drusen	***JAG1*** (Alagille syndrome 1)
Persistent foetal vasculature	***CHD7*** (CHARGE syndrome)
Retinal dysplasia, detachment	***PAX2*** (Papillorenal syndrome)
Retinal hamartoma	***TFAP2A*** (Branchiooculofacial syndrome)
**Neuro-ophthalmic abnormalities**	
Strabismus	***DHCR7*** (Smith-Lemli-Opitz syndrome); ***EYA1*** (Branchiootorenal syndrome 1, with or without cataracts); ***JAG1*** (Alagille syndrome 1); ***KMT2D*** (Kabuki syndrome 1); ***NIPBL*** (Cornelia de Lange syndrome 1); ***PBX1*** (CAKUT with or without hearing loss, abnormal ears, or developmental delay); ***SALL4*** (Duane-radial ray syndrome); ***TFAP2A*** (Branchiooculofacial syndrome)
Nystagmus	***EYA1*** (Branchiootorenal syndrome 1, with or without cataracts); ***GATA3*** (Hypoparathyroidism, sensorineural deafness, and renal dysplasia); ***NIPBL*** (Cornelia de Lange syndrome 1)
Duane anomaly (strabismus with limited horizontal eye movement)	***SALL1*** (Townes-Brocks syndrome 1; Townes-Brocks branchiootorenal-like syndrome); ***SALL4*** (Duane-radial ray syndrome)
Nocturnal lagophthalmos (eyelids do not close fully during sleep)	***KMT2D*** (Kabuki syndrome 1)

## Ocular coloboma (*CHD7, KMT2D, PAX2, PLVAP, SALL1, STRA6, TFAP2A, BMP4, CENPF, SALL4*)

Ocular coloboma affect one in 2000 live births [39], and most have a genetic basis [40].

They derive from incomplete closure of the choroidal fissure during embryonic development at about week 7.

Coloboma in CAKUT affect the iris (anterior segment), or choroid, retina and optic nerve (posterior segment). They affect the anterior and posterior segments equally often [39], and both segments are affected in about one quarter of patients. The extent of the defect depends on the stage at which the fissure fails to close, with a late failure-to-close resulting in only an iris defect [41]. Coloboma are usually unilateral but bilateral in one third of cases [39]. Ocular coloboma are also associated with developmental delay [39], and other neurological, skeletal, and craniofacial, as well as kidney anomalies [41].

Coloboma are often associated with microphthalmia, and are present from birth. The visual prognosis depends mainly on the involvement of the macula and optic nerve. Isolated coloboma of the iris do not affect vision and rarely require surgical treatment, for cosmetic reasons [42]. Chorioretinal coloboma are usually asymptomatic, require a formal ophthalmic examination for their demonstration, but may result in a visual field defect [41]. Unilateral cases may develop strabismus. Bilateral cases often present in infancy with poor vision and nystagmus.

At diagnosis, the extent of the coloboma and associated abnormalities such as microphthalmia, amblyopia, squint, and refractive errors should be identified. Direct and indirect ophthalmoscopy, accurate refraction, assessment for microphthalmia, and visual fields should be performed. Evaluation may be difficult in an infant. Individuals with a coloboma, especially of the posterior segment, require monitoring by an ophthalmologist.

In general, coloboma cannot be corrected, and their management focuses on monitoring for and treating complications such as cataract, glaucoma, retinal detachment and subretinal neovasculariation [42–46]. More than half of children with a coloboma develop another ocular abnormality including amblyopia or strabismus over a 9-year period [39]. Amblyopia may be helped with part-time occlusion, strabismus can be treated surgically and spectacles may help. Chorioretinal coloboma are associated with retinal detachments in up to 40% of affected individuals [41]. These may be overlooked because of already-limited vision. Subretinal neovascularisation at the coloboma edge may be managed with antiVEGF treatment.

The patient with CAKUT and a coloboma must also be assessed for other syndromic features, and first-degree relatives examined for coloboma, other ocular features and for CAKUT.

## Optic nerve dysplasia (*PAX2*)

Optic nerve dysplasia also described as ‘optic nerve coloboma’ or a ‘morning glory disc anomaly’ is typical of papillorenal syndrome due to *PAX2* pathogenic variants. It occurs in the majority of cases [47], is often bilateral and is characterised by the emergence of the retinal vessels from the periphery rather than the centre of the optic disc.

## Microphthalmia (*CHD7, DHCR7, FRAS1, GLI3, PAX2, SALl1, STRA6, TFAP2A, BMP4, CENPF, SALL4*)

This is a rare developmental disorder affecting one in 5000 individuals [48] where one or both eyes are abnormally small with an axial diameter less than 2SD below the mean for age. Microphthalmia may be associated with microcornea, aniridia, cataract, and retinal degeneration [49]. It usually affects vision, and treatment may be required for the more severe forms [49].

## Cataracts (*CHD7, DHCR7, EYA1, GPC3, JAG1, KMT2D, LRP4, PAX2, RET, SALL1, TFAP2A, CENPF, HS2ST1, SALL4*)

Many of these cataracts are present at birth. Again, these may be unilateral or bilateral, are detected on examination for a red reflex and those more than 3 mm in diameter affect vision. Visually-significant cataracts should be removed surgically within weeks of birth.

## Inherited retinal degeneration (*GATA3*,* NPHP3*, *PAX2*,* BMP4*)

Inherited retinal degeneration is a diverse group of diseases characterised by photoreceptor cell death and progressive loss of vision, and encompassing the retinal dystrophies, including retinitis pigmentosa. It usually reflects ciliary dysfunction which is more commonly associated with the renal ciliopathies and cystic kidney disease. The diagnosis is usually made on the basis of clinical history, fundus examination, and electroretinography. In retinitis pigmentosa, the fundus characteristically demonstrates the triad of 'bone spicules', pale optic disc, and arteriolar attenuation bilaterally.

## Hypertelorism (*WNT5A*,* CTU2*,* LRP4*,* ZMYM2*,* H2ST1*)

Hypertelorism is an abnormally-increased distance between the orbits which may be corrected surgically between the ages of 5 and 8.

## Posterior embryotoxon (*NOTCH2*, *JAG1*,* HS2ST1*) and Axenfeld anomaly (*JAG1*)

Posterior embryotoxon and Axenfeld anomaly is rare, occurring in one in 200,000 live births. It is usually diagnosed in childhood, where there is a membrane extending from the cornea onto the iris surface in both eyes. It may be associated with iris atrophy and later, glaucoma.

Peter’s anomaly (*CENPF*, *EYA1*, *NIPBL*) is where a thinned and clouded cornea attaches to the iris and results in blurred vision. It is often associated with cataracts and glaucoma.

Refractive errors (*CHD7*, *NIPBL*, *BMP4*), such as myopia and hypermetropia, correspond to short- and long-sightedness, are common, and result from defects in the shape of the eyeball, cornea, or lens.

Neuro-ophthalmic disorders (*ANOS1*, *CHRNA3*, *DHCR7*, *EYA1*, *FAM58A*, *JAG1*, *KDM6A*, *KMT2D*, *NIPBL*, *NPHP3*, *PBX1*, *SALL1*, *SALL4*) include a poor light reflex, nystagmus, and strabismus.

Duane anomaly (*FAM58A*) is a congenital anomaly where the horizontal eye movement is limited in abduction, adduction, or both. 

## Some forms of CAKUT and their ocular associations

### Papillorenal (renal-coloboma) syndrome (*PAX2*)

Papillorenal syndrome is associated with malformations of the kidneys and eyes. More than 250 affected individuals have been reported but many are unrecognised so that population frequencies are likely to be underestimates. A pathogenic variant in *PAX2* is identified in nearly 50% of cases [50]. Kidney anomalies occur in 90% of cases, with hypodysplasia in 65%, multicystic dysplasia in 10%, and vesicoureteric reflux in 14% [47, 51]. The principal ocular manifestation is a dysplastic optic nerve, which is usually bilateral [47]. This appears as an excavated optic disc, with the retinal vessels emerging at the periphery rather than centrally (Fig. 1). This is often described as an ‘optic nerve coloboma’ or ‘morning glory disc anomaly’ because of its resemblance to the flower. Detection requires a careful dilated fundus examination. Visual acuity is reduced in at least one eye in 75% of cases but may be normal. Further visual loss occurs with retinal detachment [46]. This risk necessitates close monitoring by an ophthalmologist. The ocular phenotype may differ even among family members with the same variant.

### Branchiootorenal syndrome 1 with or without cataracts (*EYA1*)

Branchiootorenal syndrome 1 is an AD-inherited disease characterised by branchial cysts or fistulae, structural defects and an abnormal shape of the outer, middle or inner ear, preauricular pits, and hearing loss [52]. It affects about one in 40,000 of the population. Kidney abnormalities including agenesis, hypoplastic and cystic kidneys, pelviureteric obstruction, bifid ureters and kidney failure occur in about two-thirds of affected individuals. The ocular associations include congenital anterior segment anomalies including cataract. However, these features are not common [14], and sometimes the kidneys are normal.

### Townes-Brocks syndrome 1 (*SALL1*)

Townes-Brocks syndrome 1 is an AD-inherited disease characterised by kidney, limb and ear anomalies as well as an imperforate anus, rectal atresia, polydactyly, and a triphalangeal thumb [50]. Other features include preauricular tags, overfolded helices, cardiac anomalies, hypospadias, and impaired kidney function. Coloboma and the Duane anomaly are rare. The features may overlap with branchiootorenal syndrome since the *EYA1* and *SALL1* genes are involved in the same biochemical pathways.

### Hypoparathyroidism, sensorineural deafness and renal dysplasia (HDR syndrome) (*GATA3*)

*GATA3* is a zinc-finger transcription factor that binds to the enhancer elements (A/T)GATA (A/G) of all four T cell antigen receptors [53], and is required for the embryonic development of the parathyroids, hearing, and kidneys. Hypoparathyroidism in HDR syndrome ranges from asymptomatic disease to features including myalgia, sensory problems, and tetany from hypocalcaemia. Parathyroid hormone levels vary from low to high. Hearing loss is obvious early. Kidney problems include developmental abnormalities such as dysplasia and hypoplasia but also cystic kidneys, vesicoureteric reflux, proteinuria, renal tubular acidosis, nephrocalcinosis, and kidney failure. Penetrance varies in different family members. Other manifestations include pyloric stenosis and female genital tract malformations [54]. Hearing loss ranges from mild to severe and is usually bilateral. The prognosis depends on disease severity. Ophthalmic features include band keratopathy and inherited retinal degeneration [55].

### CAKUT with or without hearing loss, abnormal ears or developmental delay (*PBX1*)

*PBX1* is a transcription factor that regulates morphologic patterning, organogenesis, and haematopoiesis in the embryo. It does this through modulation of the HOX protein [34]. This syndrome is sometimes referred to as CAKUTHED (Hearing loss, abnormal ears, and developmental delay). The disease usually presents in childhood. Ocular features include strabismus, corneal clouding, iris abnormalities, and glaucoma [38].

Other forms of CAKUT with interesting ocular manifestations include the following.

### CHARGE syndrome (*CHD7*)

CHARGE syndrome (Coloboma of the eye, Heart defects, nasal choanae, growth Retardation, and Genital and urinary tract abnormalities and Ear anomalies with deafness) affects one in 10,000 births [56], and results from a pathogenic variant in *CHD7* in two-thirds of cases [57]. However, these are almost all de novo so that there is often no family history [58]. Inheritance is otherwise AD and there is phenotypic variation even between affected family members. The kidney manifestations include unilateral agenesis, fusion, ectopia, and malrotation, duplex collecting systems and ureteral agenesis. The other classical features are choanal atresia, developmental delay, hypoplasia or aplasia of the semicircular canals, and cardiac defects in 80% of cases [59].

Coloboma are characteristic and seen in nearly all affected individuals [60]. They are typically chorioretinal, but may also affect the iris or optic nerve [61]. They are usually bilateral. Other reported ocular anomalies include microphthalmia and anophthalmia, microcornea, cataract, ectopia lentis, and persistent foetal vasculature [62]. There is an association with high myopia, but overall, visual acuity ranges from near normal to an absence of light perception [62]. Patients require management by an ophthalmologist, because of the risk of retinal detachment [63], and to ensure refractive errors are corrected.

### Alagille syndrome (*JAG1, NOTCH2*)

Alagille syndrome results from a pathogenic variant in *JAG1* in 97% of cases and *NOTCH2* in fewer than 1% [64]. The population frequency is one in 70,000 but this is probably an underestimate [64]. Inheritance is AD. With a *JAG1* variant, kidney anomalies occur in about 40% [65], with dysplasia being the most common, and others including vesicoureteral reflex and ureteropelvic junction obstruction [65]. Posterior embryotoxon, an anteriorly displaced and thickened Schwalbe’s line, is a cardinal feature of Alagille syndrome, occurring in 95% of cases [66]. It does not affect vision but is helpful diagnostically. Optic disc drusen are also common and usually occur bilaterally [67]. Other common ocular anomalies include those of the optic disc (76%), diffuse fundus hypopigmentation (57%), and a speckled retinal pigment epithelium (33%) [66]. The Axenfeld anomaly occurs in 13% [68].

### Rubinstein-Taybi syndrome (*CREBBP, EP300*)

Rubinstein-Taybi syndrome is characterised by short stature, microcephaly, intellectual disability, dysmorphic facial features, broad thumbs and big toes and cryptorchidism. It affects about one in 100,000 births and has AD inheritance. Pathogenic variants in *CREBBP* account for 60% of cases, *EP300* for 10% and the other genes are not known [69, 70]. Kidney malformations occur in half the cases, including kidney agenesis, duplication, hypoplasia, hydronephrosis, and vesicoureteric reflux.

More than half the affected individuals have ocular abnormalities [71]. These include strabismus (in 70%); refractive error (60%) including high myopia, in 25%; and coloboma affecting the iris, retina, choroid, or optic nerve in 10% [71–73]. Congenital and juvenile glaucoma, and congenital cataract occur [72]. Inherited retinal degeneration may be present with an abnormal retinal pigment epithelium, absent foveal reflex, and electroretinogram findings suggesting cone or cone-rod dysfunction [72], as well as peripheral retinal avascularity [74].

## Discussion

This study found that about half the genes associated with CAKUT have ocular features and that more may be expected based on the retinal expression data and the phenotypes of mouse models. There is, however, little information on the proportion of individuals with each form of CAKUT who have ocular abnormalities.

Coloboma are one of the ocular features associated with the most CAKUT genes. They are typically present from birth and do not progress during life, but complications such as amblyopia, strabismus, retinal detachment, cataract and subretinal neovascularisation may occur. Coloboma are also sometimes seen in other genetic kidney diseases such as the ciliopathies, focal and segmental glomerulosclerosis, and tubulopathies, as well as genetic diseases that do not affect the kidney, and sometimes without an obvious cause.

Many ocular abnormalities, such as coloboma, microphthalmia or strabismus, are obvious to a renal physician and should prompt a formal ophthalmological review. However, the diagnosis of CAKUT itself indicates that screening for syndromic features including an ophthalmological examination is warranted. The input from an interested ophthalmologist is important in assessment of an ocular phenotype, and in deciding on the need for active treatment or ongoing monitoring.

The demonstration of a coloboma or other ocular abnormality in a person with a structural kidney disease suggests a genetic cause, and in some cases a specific gene. However, more than 150 genes have been identified in CAKUT and the genetic detection rate is less than 20% so that testing for a variant is only advocated where a defect in a specific gene is suspected, such as for *PAX2* with optic disc dysplasia [75].

Most genes causing CAKUT demonstrate AD inheritance and first-degree family members should also be examined. However, clinical features are often incompletely penetrant and even affected family members may have no phenotype. The variable penetrance means that it is often not possible to accurately predict the kidney and visual consequences for a future child.

This study’s strengths were the use of the Genomics England CAKUT panel, and of curated retinal expression and mouse model databases to identify the ocular associations. The study’s major limitations were that individual forms of CAKUT diseases are rare, data is limited on how often ocular features occur with different forms of CAKUT, affected individuals have not necessarily undergone an ophthalmic examination, and reported ocular features may have been coincidental. The list of CAKUT genes is not exhaustive but nevertheless represents those considered by many laboratories in their search for pathogenic variants and is representative of the genes causing CAKUT and their ocular phenotypes.

Thus, clinicians should consider the possibility of ocular disease in patients with CAKUT, since these can be helpful diagnostically and may require further ophthalmic management. The ocular abnormalities in CAKUT generally do not progress over time, but complications may occur, and monitoring and treatment may be necessary.

### Supplementary Information

Below is the link to the electronic supplementary material.

Supplementary file1 (PDF 376 KB)

## Data Availability

All relevant data is included in the manuscript or in the Supplementary Information.
