# Supplementary material for: Ocular manifestations of congenital anomalies of the kidney and urinary tract (CAKUT)
Source: Pediatr Nephrol. 2023 Jul 20;39(2):357–69. doi: 10.1007/s00467-023-06068-9 (PMC10728251; doi:10.1007/s00467-023-06068-9)
Supplement: Supplementary file 1 — Supplementary file1 (PDF 376 KB) [file 467_2023_6068_MOESM1_ESM.pdf]

Suppl Table 1: CAKUT genes and their ocular associations

Gene	Disease	Renal (OMIM)	Extrarenal (OMIM)	Ocular features in humans (OMIM and references)	mRNA expression (HPA)	Mouse model (MGI)	Reference
ACE (106180)	Renal tubular dysgenesis (AR) ;267430	Renal tubular dysgenesis	Skeletal, pulmonary hypoplasia and severe hypotension	None reported	0.4 TPM	None noted	Gribouval et al. 2005 [1]; Gribouval et al. 2012 [2]
ACTG2 (102545)	Visceral myopathy (AD); 155310	Hydronephrosis, hydroureter, vesicoureteric reflux (VUR); megacysts	Absent abdominal wall musculature, pancreatitis and gastrointestinal effects	None reported	NA	No models	Thorson et al. 2013 [3]; Tobenkin 1964 [4]
AGT (106150)	Renal tubular dysgenesis (AR); 267430	Renal tubular dysgenesis	Skeletal, pulmonary hypoplasia and severe hypotension	None reported	26.4 TPM	None noted	Gribouval et al. 2005 [1]; Gribouval et al. 2012 [2]
AGTR1 (106165)	Renal tubular dysgenesis (AR); 267430	Renal tubular dysgenesis	Skeletal, pulmonary hypoplasia and severe hypotension	None reported	0.9 TPM	None noted	Gribouval et al. 2005 [1]; Gribouval et al. 2012 [2]
ANOS1 (300836)	Hypogonadotropic hypogonadism 1 with or without anosmia (Kallmann syndrome 1) (XL); 308700	Kidney agenesis	Abnormal genitalia, endocrine features	Colour blindness; abnormal eye movement, unspecified	19.6 TPM	No models	Hardelin et al. 1992 [5]; Hermanussen & Sippell 1985 [6]; Kirk et al. 1994 [7]; Massin et al. 2003 [8]; Wegenke et al. 1975 [9]
BNC2 (608669)	Lower urinary tract obstruction, congenital (AD); 618612	Kidney hypo-dysplasia; VUR; urethral stenosis, posterior urethral valve (lower urinary tract obstruction)	None reported	None reported	0.8 TPM	Increased corneal thickness	Kolvenbach et al. 2019 [10]
CEP55 (610000)	Multinucleated neurons, anhydramnios, renal dysplasia, cerebellar hypoplasia, and hydranencephaly (AR); 236500	Kidney agenesis, hypoplasia, dysplasia, cysts; ureteral agenesis; bladder agenesis	Skeletal, pulmonary hypoplasia, neurological abnormalities	None reported	NA	Cataract	Bendon et al. 1987[11]; Bondeson et al. 2017 [12]; Frosk et al. 2017 [13]; Gschwendtner et

							al. 1997 [14]; Strauss et al. 1984 [15]
CHD7 (608892)	CHARGE syndrome, (AD); 214800	Kidney agenesis, malrotation, ectopia, fusion (horseshoe kidney); hydronephrosis/ pelvic ectasia; ureteral agenesis, duplication	Skeletal, cardiac abnormalities, abdominal hernia, gastrointestinal, abnormal genitalia	High myopia, hyperopia; microphthalmia, anophthalmia, colobomatous cyst; coloboma (iris, choroid, retina, optic nerve); microcornea; ectopia lentis, cataract; persistent fetal vasculature; excavated optic nerve(“Handmann anomaly”)	24TPM	Abnormal eye morphology, small lens, abnormal optic cup, coloboma	Hittner et al. 1979 [16]; Burkitt Wright et al. 2009 [17] ; Hittner et al. 1979 [16]; Koletzko & Majewski 1984 [18]; Metlay et al. 1987 [19]; Nishina et al. 2012 [20]
CHRNA3 (118503)	Bladder dysfunction, autonomic, with impaired pupillary reflex and secondary CAKUT (AR); 191800	Kidney hypoplasia, cysts; hydronephrosis, hydroureter, VUR; thickened bladder wall	Abnormal external genitalia, orthostatic hypotension, autonomic dysfunction	Impaired pupillary light reflex (persistent miosis with poor pupillary dilation, persistent mydriasis with poor pupillary constriction)	54 TPM	Microphthalmia, impaired pupillary reflex, mydriasis , narrow eye opening	Mann et al. 2019 [21]
CTU2 (617057)	Microcephaly, facial dysmorphism, renal agenesis, and ambiguous genitalia syndrome (AR);618142	Kidney agenesis, hypoplasia, crossed fused renal ectopia	Skeletal, abnormal external genitalia, neurological, cardiac anomalies	Hypertelorism	1.4 TPM	None noted	Shaheen et al 2016 [22]
DHCR7 (602858)	Smith-Lemli-Opitz syndrome (AR);270400	Kidney agenesis, hypoplasia, dysplasia, cysts, fusion, ectopia; ureteropelvic junction obstruction, hydronephrosis	Skeletal, abnormal external genitalia, neurological, cardiac anomalies	Microphthalmia; cataract; optic atrophy, glaucoma; optic nerve hypoplasia; strabismus	8.7 TPM	Hypertelorism, strabismus, nystagmus, ptosis, cataract	Atchaneeyasakul 1998 [23]; Cotlier & Rice, 1971 [24]; Curry et al. 1987 [25]; Rutledge et al. 1984 [26]
DSTYK (612666)	Congenital anomalies of kidney and urinary tract 1 (AD); 610805	Kidney agenesis, hypoplasia, dysplasia, cysts; hydronephrosis (congenital), hydrocalyx, ureteropelvic junction obstruction, VUR; bladder agenesis	None reported	None reported	13.5 TPM	None noted	Doray et al. 1999 [27]; Sanna-Cherchi et al. 2007 [28]; Sanna-Cherchi et al. 2013 [29]
EYA1 (601653)	Branchiootorenal syndrome 1, with or	Kidney agenesis, hypoplasia, dysplasia,	Hearing loss, preauricular pits,	Nuclear-type congenital cataracts (bilateral);	0.4 TPM	Eyelids open at birth	Azuma et al. 2000 [30]; Carmi et al.

	without cataracts (AD) or Anterior segment anomalies with or without cataract; 113650	cysts, crossed ectopia, malrotation; distorted pelvicalyceal system, bifid renal pelvis, narrow ureteropelvic junction; ureteral duplication; VUR	abnormal ears, cochlear malformation, high arched palate	nystagmus, esotropia; Reduced visual acuity; central corneal opacity, Peters' anomaly; nuclear-type congenital cataract; persistent pupillary membrane (bilateral)			1983 [31]; Chitayat et al 1992 [32]; Fraser et al. 1978 [33]; Fraser et al 1983 [34]; Legius et al 1990 [35]; Melnick et al. 1975 [36]; Melnick et al. 1976 [37]
FAM58A (CCNQ) (300708)	STAR syndrome (XL);300707	Pelvic kidney, solitary kidney, hydronephrosis	Skeletal, abnormal external genitalia, cardiac anomalies	Telecanthus, Duane anomaly	6.4 TPM	None noted	Green et al, 1996 [38]
FRAS1 (607830)	Fraser syndrome 1 (AR);219000	Kidney agenesis, hypoplasia; anterior urethral atresia	Skeletal, abnormal external genitalia, facial appearance, neurological and laryngeal stenosis, hearing loss	Anophthalmia, cryptophthalmos, absent lacrimal ducts	2.2 TPM	Abnormal eye morphology, microphthalmia, narrow eye opening, eye lids open at birth	Andiran et al. 1999 [39]; Fraser 1962 [40] ; Lurie & Cherstvoy 1984 [41]; Pankau et al. 1994 [42]
FREM2 (608945)	Fraser syndrome 2 (AR); 617666	Kidney agenesis, hypodysplasia; ureteral agenesis; bladder agenesis, hypoplasia	Skeletal, abnormal external genitalia, facial appearance	Cryptophthalmos	0.3 TPM	Abnormal eye morphology, abnormal anterior eye segment, absent ciliary body, iris hypoplasia, abnormal cornea, abnormal lens , microphthalmia, anophthalmia, increased retinal thickness	Jadeja et al. 2005 [43]; Shafeghati et al. 2008 [44]
GATA3 (131320)	Hypoparathyroidism, sensorineural deafness, and renal dysplasian (AD); 146255	Kidney agenesis, hypoplasia, dysplasia; VUR	Hearing loss, abnormal female genitalia	Retinitis pigmentosa; pseudopapilledema (symmetric); horizontal nystagmus	0 TPM	Narrow eye opening	Barakat et al. 2018 [45]; Bilous et al. 1992 [46]; Ferraris et al. 2009 [47]; Hasegawa et al. 1997 [48]
GLI3 (165240)	Pallister-Hall syndrome (AD); 146510	Kidney agenesis, hypoplasia, dysplasia, ectopia, horseshoe	Skeletal, facial, cardiac, respiratory, neurological and	Microphthalmia	1.8 TPM	Anophthalmia, absent optic nerve, abnormal eye	Hall et al. 1980 [49]; Pallister et al.

		kidney; hydronephrosis, hydroureter	genitalia abnormalities			morphology, eye lids open at birth	1989 [50]; Verloes et al. 1995 [51]
GPC3 (300037)	Simpson-Golabi-Behmel syndrome, type 1 (XL); 312870	Kidney dysplasia, duplication; hydronephrosis	Skeletal, facial, cardiac, respiratory, neurological and genitalia abnormalities; hearing loss	Juvenile cataract; retinal detachment	6.2 TPM	None noted	Hughes-Benzie et al. 1992 [52]; Ireland et al. 1993 [53]; Schirwani et al. 2019 [54]
GREB1L (617782)	Renal hypodysplasia/aplasia 3 (AD); 617805	Kidney agenesis, hypoplasia, dysplasia, cysts, horseshoe kidney; hydro-nephrosis, VUR; bladder agenesis	Internal genitalia abnormalities	None reported	3.1 TPM	None noted	Brophy et al. 2017 [55]; De Tomasi et al. 2017 [56]; Sanna-Cherchi et al. 2017 [57]
GRIP1 (604597)	Fraser syndrome 3 (AR); 617667	Kidney agenesis; ureteral agenesis; bladder hypoplasia	Skeletal, facial, respiratory, gastrointestinal abnormalities	Cryptophthalmos; anterior chamber anomalies, unspecified	4.8 TPM	Abnormal eye morphology, abnormal iris, abnormal corneal thickness, corneal opacity, abnormal lens, aphakia, cataract, microphthalmia, eyelids open at birth	Vogel et al. 2012 [58]
HAAO (604521)	Vertebral, cardiac, renal and limb defects syndrome 1 (AR); 617660	Hypoplastic kidneys, reflux	Skeletal, hearing loss, neurological, cardiac anomalies, laryngeal web	None reported	0.3 TPM	None noted	Shi et al, 2017 [59]; Szot et al, 2021 [60]
HNF1B (189907)	Renal cysts and diabetes syndrome (AD);137920	Kidney agenesis, hypoplasia, dysplasia, cysts, horseshoe kidney; ureteropelvic junction obstruction	Gout, diabetes	None reported	0 TPM	None noted	Bellanne-Chantelot et al. 2004 [61]; Kaplan et al. 1989 [62]; Nakayama et al. 2010 [63]; Rizzoni et al. 1982 [64]
HOXA13 (142959)	Hand-foot-uterus syndrome (AD); 140000	Kidney failure	Skeletal abnormalities, abnormal genitalia	None reported	0 TPM	None noted	Stern 1970 [65]

HSPE2 (613469)	Urofacial syndrome 1 (AR);236730	Hydronephrosis/ dilated kidney pelvis/calyces, hydroureter; VUR, neuropathic bladder, trabeculated bladder, dilated bladder; urethral valve/obstruction	Facial, external genital anomalies	None reported	NA	No models	Daly et al. 2010 [66]; Derbent et al. 2009 [67]; Garcia-Minaur et al. 2011 [68]; Ochoa and Gorlin 1987 [69]
ITGA8 (604063)	Renal hypodysplasia/ aplasia 1 (AR); 191830	Kidney agenesis, hypoplasia, dysplasia, cysts; ureteral agenesis; bladder agenesis, hypoplasia; urethral obstruction	Facial, respiratory, skeletal anomalies	None reported	1.6 TPM	None noted	Hack et al. 1974 [70]; Schmidt et al. 1983 [71]; Selig et al. 1993 [72]; Yates et al. 1984 [73]
JAG1 (601920)	Alagille syndrome 1 (AD); 118450	Kidney hypoplasia, dysplasia, cysts; ureteropelvic junction obstruction, hydronephrosis VUR; duplicated collecting system	Facial, cardiac, skeletal, neurological, cardiac, abdominal anomalies	Myopia; microcornea, band keratopathy; posterior embryotoxon (95%), Axenfeld anomaly (13%), mosaic pattern of iris stromal hypoplasia; cataract; chorioretinal atrophy, choroidal folds, diffuse fundus hypopigmentation (57%), retinal pigment epithelium speckling (33%), peripapillary retinal depigmentation, congenital macular dystrophy; optic disc drusen (95%); ectopic pupil; strabismus	22.1 TPM	Abnormal eye morphology, abnormal iris, abnormal pupil, iris coloboma, corneal opacity, small lens, microphthalmia	Brodsky & Cunniff 1993 [74]; El-Koofy et al. 2011 [75]; Hingorani et al. 1999 [76]; Kamath et al. 2012 [77]; LaBrecque et al. 1982 [78]; Martin et al. 1996 [79]; Mueller et al. 1984 [80]; Nischal et al. 1997 [81]; Raymond et al. 1989 [82]; Shulman et al. 1984 [83]; Turnpenny & Ellard 2012 [84]
KDM6A (300128)	Kabuki syndrome (XL); 300867	Crossed fused renal ectopia, single fused kidneys	Facial, cardiac, skeletal anomalies, developmental delay	Strabismus, nystagmus	3.8 TPM	None noted	Niikawa et al, 1981 [85]
KMT2D (602113)	Kabuki syndrome 1 (AD);147290	Kidney dysplasia, cysts, fusion, crossed-fused renal ectopia;	Facial, cardiac, abdominal, external genitalia,	Myopia; blue sclerae; retinal coloboma; congenital corneal staphyloma; tilted optic disc;	48 TPM	None noted	Cheon et al. 2014 [86]; Ewart-Toland et al. 1998 [87];

		ureteropelvic junction obstruction, hydronephrosis; duplex collecting system	skeletal, neurological, endocrine anomalies	strabismus, incomplete eye closure when asleep (nocturnal lagophthalmos)			Matsumoto & Niikawa 2003 [88]; Ming et al. 2003 [89]; Tanaka et al., 2012 [90] ; Turner et al. 2005 [91]
KYNU (605197)	Vertebral, cardiac, renal and limb defects 2 (AR); 617661	Hypoplastic kidneys, solitary kidney, kidney failure	Skeletal, cardiac anomalies	None reported	1.5 TPM	None noted	Shi et al, 2021 [59]
LIFR (601559)	Stuve-Wiedemann syndrome (AR); 601559	? kidney anomalies	Facial, cardiac, skeletal anomalies	Corneal opacities, absent corneal reflexes	17.4 TPM	None noted	Stuve and Wiedemann, 1971 [92]
LRIG2 (608869)	Urofacial syndrome 2 (AR); 615112	Hydronephrosis, VUR; neurogenic bladder, trabeculated bladder	Facial appearance	None reported	6.4 TPM	None noted	Stuart et al, 2013 [93]
LRP4 (604270)	Cenani-Lenz syndactyly syndrome (AR); 212780	Kidney agenesis, hypoplasia, ectopia	Skeletal abnormalities	Congenital cataract; hypertelorism	7.9 TPM	None noted	Bacchelli et al. 2001 [94]; Jarbhou et al. 2008 [95, 96]; Li et al. 2010; Percin & Percin 2003 [97]
MYOCD (606127)	Megabladder, congenital (AD); 618719	Kidney agenesis, kidney failure	Nil else	None reported	0 TPM	None noted	Houweling et al, 2019 [98]
NADSYN1 (608285)	Vertebral, cardiac, renal, and limb defects syndrome 3 (AR); 618845	Kidney agenesis, hypoplasia; ureteral agenesis, atresia; small, tubular bladder	Cardiac and vascular anomalies; pulmonary hypoplasia, skeletal abnormalities	None reported	6.9 TPM	None noted	Szot et al, 2020 [99]
NIPBL (608667)	Cornelia de Lange syndrome 1 (AD); 122470	Kidney hypoplasia, cysts, ectopia; dilated renal pelvis; VUR	Cardiac, skeletal, respiratory and facial anomalies	High myopia; ptosis; Peters' anomaly; poor foveal reflex; nystagmus, strabismus (hypertropia), long eyelashes; optic nerve anomalies [100]	22.4 TPM	Abnormal eye morphology, abnormal eyelids, eyelids open at birth	Levin et al. 1990 [101]; Shi and Levin 2019 [102]
NOTCH2 (600275)	Alagille syndrome 2 (AD); 610205	Kidney hypoplasia, dysplasia, cysts	Cardiac, facial, liver anomalies	Posterior embryotoxon	19.3 TPM	Abnormal eye morphology, microphthalmia	McDaniell et al. 2006 [102]
NPHP3 (608002)	Meckel syndrome 7, or Renal-hepatic pancreatic	Kidney hypoplasia, dysplasia, cysts; urethral atresia	Dandy-Walker malformation,	Inherited retinal degeneration (Senior Loken syndrome),	3.1 TPM	None noted	Mollet et al, 2002 [103]

	dysplasia 1 (AR); 267010 or 208540		cardiac anomalies, liver disease	ocular motor apraxia (Cogan syndrome)			
PAX2 (167409)	Papillorenal syndrome (renal-coloboma syndrome) (AD); 120330	Kidney agenesis, hypoplasia, dysplasia, cysts, malrotation, horseshoe kidney; pyeloureteral duplication; hydronephrosis, ureteropelvic junction obstruction, ureterovesical junction obstruction, VUR	Hearing loss, joint laxity	Reduced visual acuity, visual field defects; microphthalmia, orbital cyst; coloboma (retina, optic nerve); microcornea; lens opacity, posterior lens luxation; retinal staphyloma, retinal hypoplasia, abnormal retinal pigment epithelium, pigmentary macular dysplasia, macular hyperpigmentation, chorioretinal degeneration, macular degeneration, cystic macular degeneration, retinal oedema, retinal detachment, papillomacular detachment; optic disc dysplasia, "morning glory" anomaly, excavated optic disc, optic pit, rudimentary/absent central retinal vessels, optic disc hypoplasia, aplasia, hyperplasia	1.2 TPM	Abnormal retinal vasculature, abnormal optic nerve, optic disc coloboma, abnormal retinal pigmentation, abnormal retinal nerve fibre layer	Eccles and Schimmenti, 1999 [104]; Schimmenti 2011 [105]
PBX1 (176310)	Congenital anomalies of kidney and urinary tract syndrome with or without hearing loss, abnormal ears, or developmental delay (AD); 617641	Kidney hypoplasia, dysplasia, cysts, ectopia, horseshoe kidney; dilated renal calyces/pelvis, dilated ureter; bifid ureter; VUR; urethral valve	Hearing loss ; facial cardiac anomalies, abnormal genitalia	Strabismus	17.1 TPM	Eye lids open at birth	Heidet et al. 2017 [106]; Le Tanno et al. 2017 [107]; Slavotinek et al. 2017 [108]
PLVAP (607647)	Diarrhea 10, protein-losing enteropathy type (AR); 618183	Kidney dysplasia; dilated renal pelvis, dilated ureter with kinking	Cardiac anomalies, facial anomalies, hypothyroidism	Bilateral coloboma, unspecified; multiple iris cysts	0 TPM	None noted	Broekaert et al. 2018 [109]; Elkadri et al. 2015 [110]
REN (179820)	Renal tubular dysgenesis (AR); 267430	Kidney tubular dysgenesis	Skeletal, facial anomalies	None reported	0.1 TPM	None noted	Gribouval et al. 2005 [1] ; Gribouval et al. 2012 [2]

RET (164761)	**Phaeochromocytoma (AD); 171300**	Kidney agenesis, hypoplasia, dysplasia; VUR	Hypertension, café au lait spots	Retinal angioma, congenital cataracts, hypertensive retinopathy	4.7 TPM	None noted	Jeanpierre et al. 2011 [111]; Skinner et al. 2008 [112]
ROBO2 (602431)	Vesicoureteral reflux 2 (AD); 610878	Kidney hypoplasia; VUR; duplex collecting system	None reported	None reported	15.4 TPM	None noted	Lu et al. 2007 [113]
SALL1 (602218)	Townes-Brocks syndrome 1<br><br>Townes-Brocks branchiootorenal-like syndrome (AD);107480	Kidney agenesis, hypoplasia, dysplasia, cysts, ectopia (pelvic kidney); VUR; urethral valves, stenosis	Skeletal, facial, cardiac, gastrointestinal anomalies; hearing loss	Visual impairment; microphthalmia; coloboma (iris, choroid, retina); Brushfield spots; congenital lamellar cataract; optic atrophy; Duane anomaly	4.6 TPM	None noted	Blanck et al. 2000 [114]; Botzenhart et al. 2005 [115]; Botzenhart et al. 2007 [116]; Ferraz et al. 1989 [117]; Johnson et al. 1996 [118]; Kohlhase et al. 1999 [119]; Kurnit et al. 1978 [120]; Rossmiller & Pasic 1994 [121]
SIX5 (600963)	Branchiootorenal syndrome 2 (AD): 610896	Kidney agenesis, hypoplasia, dysplasia	Facial anomalies, hearing loss	None reported	0.6 TPM	Nuclear cataract	Hoskins et al. 2007 [122]
STRA6 (610745)	Microphthalmia, syndromic 9<br><br>Microphthalmia, isolated, with coloboma 8 (AR); 601186	Kidney hypoplasia, dysplasia, ectopia (pelvic kidney), malrotation, horseshoe kidney; hydronephrosis; ureterocele	Intellectual disability; skeletal, facial, cardiac, respiratory anomalies	Microphthalmia, anophthalmia; coloboma (iris, choroid); sclerocornea; optic nerve hypoplasia/aplasia	0 TPM	Microphthalmia, abnormal retinal morphology, abnormal choroid vasculature	Berkenstadt et al. 1999 [123]; Casey et al. 2011 [124]; Li & Wei 2006 [125]; Pasutto et al. 2007 [126]; Smith et al. 1994 [127]
TBC1D1 (609850)	CAKUT	Kidney agenesis, multiple kidney cysts, vesicoureteric reflux, hydronephrosis	None reported	None reported	9.6 TPM	None noted	Kosfeld et al, 2016 [128]
TBX18 (604613)	Congenital anomalies of kidney and urinary tract 2 (AD); 143400	Kidney hypoplasia, dysplasia, duplication; ureteropelvic junction obstruction, hydronephrosis, megaureter, VUR; ureterocele	None reported	None reported	0.1 TPM	None noted	[129]Vivante et al. 2015 [130]

TFAP2A (107580)	Branchiooculofacial (BOF) syndrome (AD); 113620	Kidney agenesis, dysplasia, cysts; hydronephrosis	Hearing loss; intellectual disability, skeletal, facial anomalies	Microphthalmia, anophthalmia; coloboma (iris, retina, optic nerve); polycoria, iris pigment epithelial cyst; cataract; combined hamartoma of retina and retinal pigment epithelium; congenital strabismus	8.1 TPM	Anophthlamia, absent eyelids, aniridia, small lens, abnormal lens, abnormal cornea	Demirci et al. 2005 [129]; Fujimoto et al. 1987 [131]; Lee et al. 1982 [132]; Lin et al. 1995 [133]; Richardson et al. 1996 [134]
TMEM260 (617449)	Structural heart defects and renal anomalies syndrome (AR); 617478	Kidney cysts, kidney failure	Skeletal, cardiac and gastrointestinal anomalies	None reported	6.9 TPM	None noted	Ta-Shma et al 2017 [135]
TRAP1 (606219)	**CAKUT, VACTERL (AR); 192350**	Kidney agenesis, dysplasia, cysts, duplication; dilated renal pelvis, VUR	Vertebra, anorectal, cardiac, tracheoesophageal fistula with or without oesophageal atresia, renal malformations and limb defects	None reported	22.3 TPM	Abnormal eye morphology, unspecified	Saisawat et al. 2014 [136]
WBP11 (618083)	Vertebral, cardiac, tracheoesophageal, renal and limb defects (AD); 619227	Kidney agenesis, small kidney, pelvic kidney	Intellectual disability, skeletal, cardiac, tracheoesophageal anomalies	None reported	21.3 TPM	None noted	Martin et al, 2020 [137]
ZIC3 (300265)	VACTERL association (XL); 314390	Kidney agenesis, dysplasia, cysts, duplication, fusion; hydronephrosis; urethral atresia	Vertebra, anorectal, cardiac, tracheoesophageal fistula with or without oesophageal atresia, renal malformations and limb defects	None reported	2.7 TPM	Abnormal eye morphology, congenital cataract	Froster et al. 1996 [138]; Wessels et al. 2010 [139]; Chung et al. 2011 [140]
ZMYM2	Neurodevelopmental craniofacial syndrome with variable renal and cardiac abnormalities (AD); 619522	Kidney agenesis, hydronephrosis	Intellectual disability, skeletal, facial, cardiac anomalies	Hypertelorism	25.4 TPM	No models	Connaughton et al, 2020 [141]

Amber list							
BMP4 (112262)	Microphthalmia, syndromic 6 (AD); 607932	Kidney agenesis, hypoplasia, dysplasia, cysts; VUR	Skeletal, neurological, facial anomalies	High myopia, microphthalmia, anophthalmia; coloboma (iris, chorioretinal); retinal dystrophy; absent optic nerves	2.7 TPM	Abnormal eye morphology, abnormal iris, irregular pupil, abnormal cornea, abnormal lens, microphthalmia, abnormal retina, absent optic nerve	Bakrania et al., 2008 [142]; Bennett et al. 1991 [143]; Weber et al. 2008 [144]
CENPF (600236)	Stromme syndrome (AR); 243605	Kidney hypoplasia, dysplasia; hydronephrosis	Skeletal, cardiac, facial gastrointestinal anomalies	Astigmatism (corneal); microphthalmia; coloboma (iris, superotemporal); microcornea, sclerocornea, corneal opacity, Peters' anomaly; anterior synechiae, iris stromal hypoplasia, corectopia; cataract; tortuous retinal vessels; optic nerve hypoplasia	1.0 TPM	None noted	Bellini et al. 2002 [145]; Filges et al. 2016 [146]; Keegan et al 2004 [147]; Stromme et al. 1993 [148]; Van Bever et al. 2008 [149]; Waters et al. 2015 [150]
EXOC3L2 (616927)	Ciliopathy that resembles Meckel Gruber syndrome (AR)	Severe kidney dysplasia	Dandy-Walker malformation, developmental delay, bone marrow failure	None noted	0.1 TPM	None noted	Shaheen et al, 2016 [151]
FGF20 (605558)	Renal hypodysplasia/aplasia 2 (AR); 615721	Kidney agenesis, bilateral; ureteral agenesis	Skeletal, facial, respiratory anomalies	None reported	0.1 TPM	None noted	Barak et al. 2012 [152]
HS2ST1 (604844)	Neurofacioskeletal syndrome with or without renal agenesis (AR); 619194	Kidney agenesis	Developmental delay, skeletal and facial anomalies	Hypertelorism, myopia, iris hypoplasia, cataract, posterior embryotoxin	19.3 TPM	Abnormal eye development, cataract, iris coloboma, abnormal retina	Schneeberger et al, 2020 [153]
SALL4 (607343)	Duane-radial ray syndrome (Okihiro syndrome) (AD); 607323	Kidney agenesis, hypoplasia, malrotation, crossed ectopia with or without fusion, horseshoe kidney;	Hearing loss, skeletal, facial, cardiac, gastrointestinal	Microphthalmia; microcornea; coloboma (iris, choroid, retina, optic nerve); cataract; bilateral chorioretinal scars (between optic disc and fovea); optic	0.5 TPM	None noted	Aalfs et al. 1996 [154]; Becker et al. 2002 [155]; Ferrell et al. 1966 [156]; Halal et al. 1984 [157]

		hydronephrosis, pelvicalyceal dilatation, VUR; bladder diverticula		disc hypoplasia, dysplasia; congenital strabismus, Duane anomaly			
SLIT2 (603746)	CAKUT (AD)	Cystic dysplastic kidneys, unilateral kidney agenesis, duplicated collecting system	None reported	None reported	13.2 TPM	None noted	Hwang et al 2015 [158]
WNT5A (164975)	Robinow syndrome (AD); 180700	Kidney duplication; hydronephrosis, VUR; short urethra with anterior cystic dilatation	Intellectual disability, skeletal, facial, cardiac anomalies	Hypertelorism	1.2 TPM	None noted	Bain et al. 1986 [159]; Roifman et al. 2015 [160]; Turken et al. 1996 [161]

References

1. Gribouval O, Gonzales M, Neuhaus T, Aziza J, Bieth E, Laurent N, Bouton JM, Feuillet F, Makni S, Ben Amar H, Laube G, Delezoide AL, Bouvier R, Dijoud F, Ollagnon-Roman E, Roume J, Joubert M, Antignac C, Gubler MC (2005) Mutations in genes in the renin-angiotensin system are associated with autosomal recessive renal tubular dysgenesis. Nat Genet 37:964-968.
2. Gribouval O, Moriniere V, Pawtowski A, Arrondel C, Sallinen SL, Saloranta C, Clericuzio C, Viot G, Tantau J, Blesson S, Cloarec S, Machet MC, Chitayat D, Thauvin C, Laurent N, Sampson JR, Bernstein JA, Clemenson A, Prieur F, Daniel L, Levy-Mozziconacci A, Lachlan K, Alessandri JL, Cartault F, Riviere JP, Picard N, Baumann C, Delezoide AL, Belar Ortega M, Chassaing N, Labrune P, Yu S, Firth H, Wellesley D, Bitzan M, Alfares A, Braverman N, Krogh L, Tolmie J, Gaspar H, Doray B, Majore S, Bonneau D, Triau S, Loirat C, David A, Bartholdi D, Peleg A, Brackman D, Stone R, DeBerardinis R, Corvol P, Michaud A, Antignac C, Gubler MC (2012) Spectrum of mutations in the renin-angiotensin system genes in autosomal recessive renal tubular dysgenesis. Hum Mutat 33:316-326.
3. Thorson W, Diaz-Horta O, Foster J, 2nd, Spiliopoulos M, Quintero R, Farooq A, Blanton S, Tekin M (2014) De novo ACTG2 mutations cause congenital distended bladder, microcolon, and intestinal hypoperistalsis. Hum Genet 133:737-742.
4. Tobenkin MI (1964) Hereditary Vesicoureteral Reflux. South Med J 57:139-147.
5. Hardelin JP, Levilliers J, del Castillo I, Cohen-Salmon M, Legouis R, Blanchard S, Compain S, Bouloux P, Kirk J, Moraine C, et al. (1992) X chromosome-linked Kallmann syndrome: stop mutations validate the candidate gene. Proc Natl Acad Sci U S A 89:8190-8194.
6. Hermanussen M, Sippell WG (1985) Heterogeneity of Kallmann's syndrome. Clin Genet 28:106-111.
7. Kirk JM, Grant DB, Besser GM, Shalet S, Quinton R, Smith CS, White M, Edwards O, Bouloux PM (1994) Unilateral renal aplasia in X-linked Kallmann's syndrome. Clin Genet 46:260-262.
8. Massin N, Pecheux C, Eloit C, Bensimon JL, Galey J, Kuttenn F, Hardelin JP, Dode C, Touraine P (2003) X chromosome-linked Kallmann syndrome: clinical heterogeneity in three siblings carrying an intragenic deletion of the KAL-1 gene. J Clin Endocrinol Metab 88:2003-2008.
9. Wegenke JD, Uehling DT, Wear JB, Jr., Gordon ES, Bargman JG, Deacon JS, Herrmann JP, Opitz JM (1975) Familial Kallmann syndrome with unilateral renal aplasia. Clin Genet 7:368-381.
10. Kolvenbach CM, Dworschak GC, Frese S, Japp AS, Schuster P, Wenzlitschke N, Yilmaz O, Lopes FM, Pryalukhin A, Schierbaum L, van der Zanden LFM, Kause F, Schneider R, Taranta-Janusz K, Szczepanska M, Pawlaczyk K, Newman WG, Beaman GM, Stuart HM, Cervellione RM, Feitz WFJ, van Rooij I, Schreuder MF, Steffens M, Weber S, Merz WM, Feldkotter M, Hoppe B, Thiele H, Altmuller J, Berg C, Kristiansen G, Ludwig M, Reutter H, Woolf AS, Hildebrandt F, Grote P, Zaniew M, Odermatt B, Hilger AC (2019) Rare Variants in BNC2 Are Implicated in Autosomal-Dominant Congenital Lower Urinary-Tract Obstruction. Am J Hum Genet 104:994-1006.
11. Bendon RW, Siddiqi T, de Courten-Myers G, Dignan P (1987) Recurrent developmental anomalies: 1. Syndrome of hydranencephaly with renal aplastic dysplasia; 2. Polyvalvular developmental heart defect. Am J Med Genet Suppl 3:357-365.
12. Bondeson ML, Ericson K, Gudmundsson S, Ameur A, Ponten F, Wesstrom J, Frykholm C, Wilbe M (2017) A nonsense mutation in CEP55 defines a new locus for a Meckel-like syndrome, an autosomal recessive lethal fetal ciliopathy. Clin Genet 92:510-516.
13. Frosk P, Arts HH, Philippe J, Gunn CS, Brown EL, Chodirker B, Simard L, Majewski J, Fahiminiya S, Russell C, Liu YP, Consortium FC, Canadian Rare Diseases M, Mechanisms N, Hegele R, Katsanis N, Goerz C, Del Bigio MR, Davis EE (2017) A truncating mutation in CEP55 is the likely cause of MARCH, a novel syndrome affecting neuronal mitosis. J Med Genet 54:490-501.

14. Gschwendtner A, Mairinger T, Soelder E, Alge A, Kreczy A (1997) Hydranencephaly with renal dysgenesis: a coincidental finding? Case report with review of the literature. Gynecol Obstet Invest 44:206-210.
15. Strauss S, Bouzouki M, Goldfarb H, Uppal V, Costales F (1984) Antenatal ultrasound diagnosis of an unusual case of hydranencephaly. J Clin Ultrasound 12:420-422.
16. Hittner HM, Hirsch NJ, Kreh GM, Rudolph AJ (1979) Colobomatous microphthalmia, heart disease, hearing loss, and mental retardation--a syndrome. J Pediatr Ophthalmol Strabismus 16:122-128.
17. Wright EM, O'Connor R, Kerr BA (2009) Radial aplasia in CHARGE syndrome: a new association. Eur J Med Genet 52:239-241.
18. Koletzko B, Majewski F (1984) Congenital anomalies in patients with choanal atresia: CHARGE-association. Eur J Pediatr 142:271-275.
19. Metlay LA, Smythe PS, Miller ME (1987) Familial CHARGE syndrome: clinical report with autopsy findings. Am J Med Genet 26:577-581.
20. Nishina S, Kosaki R, Yagihashi T, Azuma N, Okamoto N, Hatsukawa Y, Kurosawa K, Yamane T, Mizuno S, Tsuzuki K, Kosaki K (2012) Ophthalmic features of CHARGE syndrome with CHD7 mutations. Am J Med Genet A 158A:514-518.
21. Mann N, Kause F, Henze EK, Gharpure A, Shril S, Connaughton DM, Nakayama M, Klambt V, Majmundar AJ, Wu CW, Kolvenbach CM, Dai R, Chen J, van der Ven AT, Ityel H, Tooley MJ, Kari JA, Bownass L, El Desoky S, De Franco E, Shalaby M, Tasic V, Bauer SB, Lee RS, Beckel JM, Yu W, Mane SM, Lifton RP, Reutter H, Ellard S, Hibbs RE, Kawate T, Hildebrandt F (2019) CAKUT and Autonomic Dysfunction Caused by Acetylcholine Receptor Mutations. Am J Hum Genet 105:1286-1293.
22. Shaheen R, Al-Salam Z, El-Hattab AW, Alkuraya FS (2016) The syndrome dysmorphic facies, renal agenesis, ambiguous genitalia, microcephaly, polydactyly and lissencephaly (DREAM-PL): Report of two additional patients. Am J Med Genet A 170:3222-3226.
23. Atchaneeyasakul LO, Linck LM, Connor WE, Weleber RG, Steiner RD (1998) Eye findings in 8 children and a spontaneously aborted fetus with RSH/Smith-Lemli-Opitz syndrome. Am J Med Genet 80:501-505.
24. Cotlier E, Rice P (1971) Cataracts in the Smith-Lemli-Opitz syndrome. Am J Ophthalmol 72:955-959.
25. Curry CJ, Carey JC, Holland JS, Chopra D, Fineman R, Golabi M, Sherman S, Pagon RA, Allanson J, Shulman S, et al. (1987) Smith-Lemli-Opitz syndrome-type II: multiple congenital anomalies with male pseudohermaphroditism and frequent early lethality. Am J Med Genet 26:45-57.
26. Rutledge JC, Friedman JM, Harrod MJ, Currarino G, Wright CG, Pinckney L, Chen H (1984) A "new" lethal multiple congenital anomaly syndrome: joint contractures, cerebellar hypoplasia, renal hypoplasia, urogenital anomalies, tongue cysts, shortness of limbs, eye abnormalities, defects of the heart, gallbladder agenesis, and ear malformations. Am J Med Genet 19:255-264.
27. Doray B, Gasser B, Reinartz I, Stoll C (1999) Hereditary renal adysplasia in a three generations family. Genet Couns 10:251-257.
28. Sanna-Cherchi S, Caridi G, Weng PL, Scolari F, Perfumo F, Gharavi AG, Ghiggeri GM (2007) Genetic approaches to human renal agenesis/hypoplasia and dysplasia. Pediatr Nephrol 22:1675-1684.
29. Sanna-Cherchi S, Sampogna RV, Papeta N, Burgess KE, Nees SN, Perry BJ, Choi M, Bodria M, Liu Y, Weng PL, Lozanovski VJ, Verbitsky M, Lugani F, Sterken R, Paragas N, Caridi G, Carrea A, Dagnino M, Materna-Kiryluk A, Santamaria G, Murtas C, Ristoska-Bojkovska N, Izzi C, Kacak N, Bianco B, Giberti S, Gigante M, Piaggio G, Gesualdo L, Vukic DK, Vukojevic K, Saraga-Babic M, Saraga M, Gucev Z, Allegri L, Latos-Bielenska A, Casu D, State M, Scolari F, Ravazzolo R, Kiryluk K, Al-Awqati Q, D'Agati VD, Drummond IA, Tasic V, Lifton RP, Ghiggeri GM, Gharavi AG (2013) Mutations in DSTYK and dominant urinary tract malformations. N Engl J Med 369:621-629.

30. Azuma N, Hirakiyama A, Inoue T, Asaka A, Yamada M (2000) Mutations of a human homologue of the Drosophila eyes absent gene (EYA1) detected in patients with congenital cataracts and ocular anterior segment anomalies. Hum Mol Genet 9:363-366.
31. Carmi R, Binshtock M, Abeliovich D, Bar-Ziv J (1983) The branchio-oto-renal (BOR) syndrome: report of bilateral renal agenesis in three sibs. Am J Med Genet 14:625-627.
32. Chitayat D, Hodgkinson KA, Chen MF, Haber GD, Nakishima S, Sando I (1992) Branchio-oto-renal syndrome: further delineation of an underdiagnosed syndrome. Am J Med Genet 43:970-975.
33. Fraser FC, Ling D, Clogg D, Nogrady B (1978) Genetic aspects of the BOR syndrome--branchial fistulas, ear pits, hearing loss, and renal anomalies. Am J Med Genet 2:241-252.
34. Fraser FC, Ayme S, Halal F, Sproule J (1983) Autosomal dominant duplication of the renal collecting system, hearing loss, and external ear anomalies: a new syndrome? Am J Med Genet 14:473-478.
35. Legius E, Fryns JP, Van den Berghe H (1990) Dominant branchial cleft syndrome with characteristics of both branchio-oto-renal and branchio-oculo-facial syndrome. Clin Genet 37:347-350.
36. Melnick M, Bixler D, Silk K, Yune H, Nance WE (1975) Autosomal dominant branchiootorenal dysplasia. Birth Defects Orig Artic Ser 11:121-128.
37. Melnick M, Bixler D, Nance WE, Silk K, Yune H (1976) Familial branchio-oto-renal dysplasia: a new addition to the branchial arch syndromes. Clin Genet 9:25-34.
38. Green AJ, Sandford RN, Davison BC (1996) An autosomal dominant syndrome of renal and anogenital malformations with syndactyly. J Med Genet 33:594-596.
39. Andiran F, Tanyel FC, Hicsonmez A (1999) Fraser syndrome associated with anterior urethral atresia. Am J Med Genet 82:359-361.
40. Fraser GR (1962) XX chromosomes and renal agenesis Lancet 297:1427.
41. Lurie IW, Cherstvoy ED (1984) Renal agenesis as a diagnostic feature of the cryptophthalmos-syndactyly syndrome. Clin Genet 25:528-532.
42. Pankau R, Partsch CJ, Janig U, Meinecke R (1994) Fraser (Cryptophthalmos-syndactyly) syndrome: a case with bilateral anophthalmia but presence of normal eyelids. Genet Couns 5:191-194.
43. Jadeja S, Smyth I, Pitera JE, Taylor MS, van Haelst M, Bentley E, McGregor L, Hopkins J, Chalepakis G, Philip N, Perez Aytes A, Watt FM, Darling SM, Jackson I, Woolf AS, Scambler PJ (2005) Identification of a new gene mutated in Fraser syndrome and mouse myelencephalic blebs. Nat Genet 37:520-525.
44. Shafeghati Y, Kniepert A, Vakili G, Zenker M (2008) Fraser syndrome due to homozygosity for a splice site mutation of FREM2. Am J Med Genet A 146A:529-531.
45. Barakat AJ, Raygada M, Rennert OM (2018) Barakat syndrome revisited. Am J Med Genet A 176:1341-1348.
46. Bilous RW, Murty G, Parkinson DB, Thakker RV, Coulthard MG, Burn J, Mathias D, Kendall-Taylor P (1992) Brief report: autosomal dominant familial hypoparathyroidism, sensorineural deafness, and renal dysplasia. N Engl J Med 327:1069-1074.
47. Ferraris S, Del Monaco AG, Garelli E, Carando A, De Vito B, Pappi P, Lala R, Ponzone A (2009) HDR syndrome: a novel "de novo" mutation in GATA3 gene. Am J Med Genet A 149A:770-775.
48. Hasegawa T, Hasegawa Y, Aso T, Koto S, Nagai T, Tsuchiya Y, Kim KC, Ohashi H, Wakui K, Fukushima Y (1997) HDR syndrome (hypoparathyroidism, sensorineural deafness, renal dysplasia) associated with del(10)(p13). Am J Med Genet 73:416-418.
49. Hall JG, Pallister PD, Clarren SK, Beckwith JB, Wiglesworth FW, Fraser FC, Cho S, Benke PJ, Reed SD (1980) Congenital hypothalamic hamartoblastoma, hypopituitarism, imperforate anus and postaxial polydactyly--a new syndrome? Part I: clinical, causal, and pathogenetic considerations. Am J Med Genet 7:47-74.

50. Pallister PD, Hecht F, Herrman J (1989) Three additional cases of the congenital hypothalamic "hamartoblastoma" (Pallister-Hall) syndrome. Am J Med Genet 33:500-501.
51. Verloes A, Narcy F, Fallet-Bianco C (1995) Syndromal hypothalamic hamartoblastoma with holoprosencephaly sequence, microphthalmia, pulmonary malformations, radial hypoplasia and mullerian regression: further delineation of a new syndrome? Clin Dysmorphol 4:33-37.
52. Hughes-Benzie RM, Hunter AG, Allanson JE, Mackenzie AE (1992) Simpson-Golabi-Behmel syndrome associated with renal dysplasia and embryonal tumor: localization of the gene to Xqcen-q21. Am J Med Genet 43:428-435.
53. M I, R H-B, J A, A B, A M, J B (1993) Simpson-Golabi-Behmel syndrome in a 5 generation family: a clinical and molecular study. Proc Greenwood Center 12:41-44.
54. Schirwani S, Novelli A, Digilio MC, Bourn D, Wilson V, Roberts C, Dallapiccola B, Hobson E (2019) Duplications of GPC3 and GPC4 genes in symptomatic female carriers of Simpson-Golabi-Behmel syndrome type 1. Eur J Med Genet 62:243-247.
55. Brophy PD, Rasmussen M, Parida M, Bonde G, Darbro BW, Hong X, Clarke JC, Peterson KA, Denegre J, Schneider M, Sussman CR, Sunde L, Lildballe DL, Hertz JM, Cornell RA, Murray SA, Manak JR (2017) A Gene Implicated in Activation of Retinoic Acid Receptor Targets Is a Novel Renal Agenesis Gene in Humans. Genetics 207:215-228.
56. De Tomasi L, David P, Humbert C, Silbermann F, Arrondel C, Tores F, Fouquet S, Desgrange A, Niel O, Bole-Feysot C, Nitschke P, Roume J, Cordier MP, Pietrement C, Isidor B, Khau Van Kien P, Gonzales M, Saint-Frison MH, Martinovic J, Novo R, Piard J, Cabrol C, Verma IC, Puri R, Journel H, Aziza J, Gavard L, Said-Menthon MH, Heidet L, Saunier S, Jeanpierre C (2017) Mutations in GREB1L Cause Bilateral Kidney Agenesis in Humans and Mice. Am J Hum Genet 101:803-814.
57. Sanna-Cherchi S, Khan K, Westland R, Krithivasan P, Fievet L, Rasouly HM, Ionita-Laza I, Capone VP, Fasel DA, Kiryluk K, Kamalakaran S, Bodria M, Otto EA, Sampson MG, Gillies CE, Vega-Warner V, Vukojevic K, Pediaditakis I, Makar GS, Mitrotti A, Verbitsky M, Martino J, Liu Q, Na YJ, Goj V, Ardissino G, Gigante M, Gesualdo L, Janezcko M, Zaniew M, Mendelsohn CL, Shril S, Hildebrandt F, van Wijk JAE, Arapovic A, Saraga M, Allegri L, Izzi C, Scolari F, Tasic V, Ghiggeri GM, Latos-Bielenska A, Materna-Kiryluk A, Mane S, Goldstein DB, Lifton RP, Katsanis N, Davis EE, Gharavi AG (2017) Exome-wide Association Study Identifies GREB1L Mutations in Congenital Kidney Malformations. Am J Hum Genet 101:789-802.
58. Vogel MJ, van Zon P, Brueton L, Gijzen M, van Tuil MC, Cox P, Schanze D, Kariminejad A, Ghaderi-Sohi S, Blair E, Zenker M, Scambler PJ, Ploos van Amstel HK, van Haelst MM (2012) Mutations in GRIP1 cause Fraser syndrome. J Med Genet 49:303-306.
59. Shi H, Enriquez A, Rapadas M, Martin E, Wang R, Moreau J, Lim CK, Szot JO, Ip E, Hughes JN, Sugimoto K, Humphreys DT, McInerney-Leo AM, Leo PJ, Maghzal GJ, Halliday J, Smith J, Colley A, Mark PR, Collins F, Sillence DO, Winlaw DS, Ho JWK, Guillemin GJ, Brown MA, Kikuchi K, Thomas PQ, Stocker R, Giannoulatou E, Chapman G, Duncan EL, Sparrow DB, Dunwoodie SL (2017) NAD Deficiency, Congenital Malformations, and Niacin Supplementation. N Engl J Med 377:544-552.
60. Szot JO, Slavotinek A, Chong K, Brandau O, Nezarati M, Cueto-Gonzalez AM, Patel MS, Devine WP, Rego S, Acyinena AP, Shannon P, Myles-Reid D, Blaser S, Mieghem TV, Yavuz-Kienle H, Skladny H, Miller K, Riera MDT, Martinez SA, Tizzano EF, Dupuis L, James Stavropoulos D, McNiven V, Mendoza-Londono R, Elliott AM, Study C, Phillips RS, Chapman G, Dunwoodie SL (2021) New cases that expand the genotypic and phenotypic spectrum of Congenital NAD Deficiency Disorder. Hum Mutat 42:862-876.
61. Bellanne-Chantelot C, Chauveau D, Gautier JF, Dubois-Laforgue D, Clauin S, Beaufils S, Wilhelm JM, Boitard C, Noel LH, Velho G, Timsit J (2004) Clinical spectrum associated with hepatocyte nuclear factor-1beta mutations. Ann Intern Med 140:510-517.
62. Kaplan BS, Gordon I, Pincott J, Barratt TM (1989) Familial hypoplastic glomerulocystic kidney disease: a definite entity with dominant inheritance. Am J Med Genet 34:569-573.

63. Nakayama M, Nozu K, Goto Y, Kamei K, Ito S, Sato H, Emi M, Nakanishi K, Tsuchiya S, Iijima K (2010) HNF1B alterations associated with congenital anomalies of the kidney and urinary tract. Pediatr Nephrol 25:1073-1079.
64. Rizzoni G, Loirat C, Levy M, Milanesi C, Zachello G, Mathieu H (1982) Familial hypoplastic glomerulocystic kidney. A new entity? Clin Nephrol 18:263-268.
65. Stern AM, Gall JC, Jr., Perry BL, Stimson CW, Weitkamp LR, Poznanski AK (1970) The hand-food-uterus syndrome: a new hereditary disorder characterized by hand and foot dysplasia, dermatoglyphic abnormalities, and partial duplication of the female genital tract. J Pediatr 77:109-116.
66. Daly SB, Urquhart JE, Hilton E, McKenzie EA, Kammerer RA, Lewis M, Kerr B, Stuart H, Donnai D, Long DA, Burgu B, Aydogdu O, Derbent M, Garcia-Minaur S, Reardon W, Gener B, Shalev S, Smith R, Woolf AS, Black GC, Newman WG (2010) Mutations in HPSE2 cause urofacial syndrome. Am J Hum Genet 86:963-969.
67. Derbent M, Melek E, Arman A, Uckan S, Baskin E (2009) Urofacial (ochoa) syndrome: can a facial gestalt represent severe voiding dysfunction? Ren Fail 31:589-592.
68. Garcia-Minaur S, Oliver F, Yanez JM, Soriano JR, Quinn F, Reardon W (2001) Three new European cases of urofacial (Ochoa) syndrome. Clin Dysmorphol 10:165-170.
69. Ochoa B, Gorlin RJ (1987) Urofacial (ochoa) syndrome. Am J Med Genet 27:661-667.
70. Hack M, Jaffe J, Blankstein J, Goodman RM, Brish M (1974) Familial aggregation in bilateral renal agenesis. Clin Genet 5:173-177.
71. Schmidt W, Schroeder TM, Buchinger G, Kubli F (1982) Genetics, pathoanatomy and prenatal diagnosis of Potter I syndrome and other urogenital tract diseases. Clin Genet 22:105-127.
72. Selig AM, Benacerraf B, Greene MF, Garber MF, Genest DR (1993) Renal dysplasia, megalocystis, and sirenomelia in four siblings. Teratology 47:65-71.
73. Yates JR, Mortimer G, Connor JM, Duke JE (1984) Concordant monozygotic twins with bilateral renal agenesis. J Med Genet 21:66-67.
74. Brodsky MC, Cunniff C (1993) Ocular anomalies in the alagille syndrome (arteriohepatic dysplasia). Ophthalmology 100:1767-1774.
75. El-Koofy NM, El-Mahdy R, Fahmy ME, El-Hennawy A, Farag MY, El-Karaksy HM (2011) Alagille syndrome: clinical and ocular pathognomonic features. Eur J Ophthalmol 21:199-206.
76. Hingorani M, Nischal KK, Davies A, Bentley C, Vivian A, Baker AJ, Mieli-Vergani G, Bird AC, Aclimandos WA (1999) Ocular abnormalities in Alagille syndrome. Ophthalmology 106:330-337.
77. Kamath BM, Podkameni G, Hutchinson AL, Leonard LD, Gerfen J, Krantz ID, Piccoli DA, Spinner NB, Loomes KM, Meyers K (2012) Renal anomalies in Alagille syndrome: a disease-defining feature. Am J Med Genet A 158A:85-89.
78. LaBrecque DR, Mitros FA, Nathan RJ, Romanchuk KG, Judisch GF, El-Khoury GH (1982) Four generations of arteriohepatic dysplasia. Hepatology 2:467-474.
79. Martin SR, Garel L, Alvarez F (1996) Alagille's syndrome associated with cystic renal disease. Arch Dis Child 74:232-235.
80. Mueller RF, Pagon RA, Pepin MG, Haas JE, Kawabori I, Stevenson JG, Stephan MJ, Blumhagen JD, Christie DL (1984) Arteriohepatic dysplasia: phenotypic features and family studies. Clin Genet 25:323-331.
81. Nischal KK, Hingorani M, Bentley CR, Vivian AJ, Bird AC, Baker AJ, Mowat AP, Mieli-Vergani G, Aclimandos WA (1997) Ocular ultrasound in Alagille syndrome: a new sign. Ophthalmology 104:79-85.
82. Raymond WR, Kearney JJ, Parmley VC (1989) Ocular findings in arteriohepatic dysplasia (Alagille's syndrome). Arch Ophthalmol 107:1077.
83. Shulman SA, Hyams JS, Gunta R, Greenstein RM, Cassidy SB (1984) Arteriohepatic dysplasia (Alagille syndrome): extreme variability among affected family members. Am J Med Genet 19:325-332.

84. Turnpenny PD, Ellard S (2012) Alagille syndrome: pathogenesis, diagnosis and management. Eur J Hum Genet 20:251-257.
85. Niikawa N, Matsuura N, Fukushima Y, Ohsawa T, Kajii T (1981) Kabuki make-up syndrome: a syndrome of mental retardation, unusual facies, large and protruding ears, and postnatal growth deficiency. J Pediatr 99:565-569.
86. Cheon CK, Sohn YB, Ko JM, Lee YJ, Song JS, Moon JW, Yang BK, Ha IS, Bae EJ, Jin HS, Jeong SY (2014) Identification of KMT2D and KDM6A mutations by exome sequencing in Korean patients with Kabuki syndrome. J Hum Genet 59:321-325.
87. Ewart-Toland A, Enns GM, Cox VA, Mohan GC, Rosenthal P, Golabi M (1998) Severe congenital anomalies requiring transplantation in children with Kabuki syndrome. Am J Med Genet 80:362-367.
88. Matsumoto N, Niikawa N (2003) Kabuki make-up syndrome: a review. Am J Med Genet C Semin Med Genet 117C:57-65.
89. Ming JE, Russell KL, Bason L, McDonald-McGinn DM, Zackai EH (2003) Coloboma and other ophthalmologic anomalies in Kabuki syndrome: distinction from charge association. Am J Med Genet A 123A:249-252.
90. Tanaka R, Takenouchi T, Uchida K, Sato T, Fukushima H, Yoshihashi H, Takahashi T, Tsubota K, Kosaki K (2012) Congenital corneal staphyloma as a complication of Kabuki syndrome. Am J Med Genet A 158A:2000-2002.
91. Turner C, Lachlan K, Amerasinghe N, Hodgkins P, Maloney V, Barber J, Temple IK (2005) Kabuki syndrome: new ocular findings but no evidence of 8p22-p23.1 duplications in a clinically defined cohort. Eur J Hum Genet 13:716-720.
92. Stuve A, Wiedemann HR (1971) Congenital bowing of the long bones in two sisters. Lancet 2:495.
93. Stuart HM, Roberts NA, Burgu B, Daly SB, Urquhart JE, Bhaskar S, Dickerson JE, Mermerkaya M, Silay MS, Lewis MA, Olondriz MB, Gener B, Beetz C, Varga RE, Gulpinar O, Suer E, Soygur T, Ozcakar ZB, Yalcinkaya F, Kavaz A, Bulum B, Gucuk A, Yue WW, Erdogan F, Berry A, Hanley NA, McKenzie EA, Hilton EN, Woolf AS, Newman WG (2013) LRIG2 mutations cause urofacial syndrome. Am J Hum Genet 92:259-264.
94. Bacchelli C, Goodman FR, Scambler PJ, Winter RM (2001) Cenani-Lenz syndrome with renal hypoplasia is not linked to FORMIN or GREMLIN. Clin Genet 59:203-205.
95. Jarbhou H, Hamamy H, Al-Hadidy A, Ajlouni K (2008) Cenani-Lenz syndactyly with facial dysmorphism, hypothyroidism, and renal hypoplasia: a case report. Clin Dysmorphol 17:269-270.
96. Li Y, Pawlik B, Elcioglu N, Aglan M, Kayserili H, Yigit G, Percin F, Goodman F, Nurnberg G, Cenani A, Urquhart J, Chung BD, Ismail S, Amr K, Aslanger AD, Becker C, Netzer C, Scambler P, Eyaid W, Hamamy H, Clayton-Smith J, Hennekam R, Nurnberg P, Herz J, Temtamy SA, Wollnik B (2010) LRP4 mutations alter Wnt/beta-catenin signaling and cause limb and kidney malformations in Cenani-Lenz syndrome. Am J Hum Genet 86:696-706.
97. Percin EF, Percin S (2003) Two unusual types of syndactyly in the same family; Cenani-Lenz type and "new" type versus severe type I syndactyly? Genet Couns 14:313-319.
98. Houweling AC, Beaman GM, Postma AV, Gainous TB, Lichtenbelt KD, Brancati F, Lopes FM, van der Made I, Polstra AM, Robinson ML, Wright KD, Ellingford JM, Jackson AR, Overwater E, Genesio R, Romano S, Camerota L, D'Angelo E, Meijers-Heijboer EJ, Christoffels VM, McHugh KM, Black BL, Newman WG, Woolf AS, Creemers EE (2019) Loss-of-function variants in myocardin cause congenital megabladder in humans and mice. J Clin Invest 129:5374-5380.
99. Szot JO, Campagnolo C, Cao Y, Iyer KR, Cuny H, Drysdale T, Flores-Daboub JA, Bi W, Westerfield L, Liu P, Leung TN, Choy KW, Chapman G, Xiao R, Siu VM, Dunwoodie SL (2020) Bi-allelic Mutations in NADSYN1 Cause Multiple Organ Defects and Expand the Genotypic Spectrum of Congenital NAD Deficiency Disorders. Am J Hum Genet 106:129-136.

100. McDaniell R, Warthen DM, Sanchez-Lara PA, Pai A, Krantz ID, Piccoli DA, Spinner NB (2006) NOTCH2 mutations cause Alagille syndrome, a heterogeneous disorder of the notch signaling pathway. Am J Hum Genet 79:169-173.
101. Levin AV, Seidman DJ, Nelson LB, Jackson LG (1990) Ophthalmologic findings in the Cornelia de Lange syndrome. J Pediatr Ophthalmol Strabismus 27:94-102.
102. Shi A, Levin AV (2019) Ophthalmologic findings in the Cornelia de Lange syndrome. Ophthalmic Genet 40:1-6.
103. Mollet G, Salomon R, Gribouval O, Silbermann F, Bacq D, Landthaler G, Milford D, Nayir A, Rizzoni G, Antignac C, Saunier S (2002) The gene mutated in juvenile nephronophthisis type 4 encodes a novel protein that interacts with nephrocystin. Nat Genet 32:300-305.
104. Eccles MR, Schimmenti LA (1999) Renal-coloboma syndrome: a multi-system developmental disorder caused by PAX2 mutations. Clin Genet 56:1-9.
105. Schimmenti LA (2011) Renal coloboma syndrome. Eur J Hum Genet 19:1207-1212.
106. Heidet L, Moriniere V, Henry C, De Tomasi L, Reilly ML, Humbert C, Alibeu O, Fourrage C, Bole-Feysot C, Nitschke P, Tores F, Bras M, Jeanpierre M, Pietrement C, Gaillard D, Gonzales M, Novo R, Schaefer E, Roume J, Martinovic J, Malan V, Salomon R, Saunier S, Antignac C, Jeanpierre C (2017) Targeted Exome Sequencing Identifies PBX1 as Involved in Monogenic Congenital Anomalies of the Kidney and Urinary Tract. J Am Soc Nephrol 28:2901-2914.
107. Le Tanno P, Breton J, Bidart M, Satre V, Harbuz R, Ray PF, Bosson C, Dieterich K, Jaillard S, Odent S, Poke G, Beddow R, Digilio MC, Novelli A, Bernardini L, Pisanti MA, Mackenroth L, Hackmann K, Vogel I, Christensen R, Fokstuen S, Bena F, Amblard F, Devillard F, Vieville G, Apostolou A, Jouk PS, Guebre-Egziabher F, Sartelet H, Coutton C (2017) PBX1 haploinsufficiency leads to syndromic congenital anomalies of the kidney and urinary tract (CAKUT) in humans. J Med Genet 54:502-510.
108. Slavotinek A, Risolino M, Losa M, Cho MT, Monaghan KG, Schneidman-Duhovny D, Parisotto S, Herkert JC, Stegmann APA, Miller K, Shur N, Chui J, Muller E, DeBrosse S, Szot JO, Chapman G, Pachter NS, Winlaw DS, Mendelsohn BA, Dalton J, Sarafoglou K, Karachunski PI, Lewis JM, Pedro H, Dunwoodie SL, Selleri L, Shieh J (2017) De novo, deleterious sequence variants that alter the transcriptional activity of the homeoprotein PBX1 are associated with intellectual disability and pleiotropic developmental defects. Hum Mol Genet 26:4849-4860.
109. Broekaert IJ, Becker K, Gottschalk I, Korber F, Dotsch J, Thiele H, Altmuller J, Nurnberg P, Hunseler C, Cirak S (2018) Mutations in plasmalemma vesicle-associated protein cause severe syndromic protein-losing enteropathy. J Med Genet 55:637-640.
110. Elkadri A, Thoeni C, Deharvengt SJ, Murchie R, Guo C, Stavropoulos JD, Marshall CR, Wales P, Bandsma R, Cutz E, Roifman CM, Chitayat D, Avitzur Y, Stan RV, Muise AM (2015) Mutations in Plasmalemma Vesicle Associated Protein Result in Sieving Protein-Losing Enteropathy Characterized by Hypoproteinemia, Hypoalbuminemia, and Hypertriglyceridemia. Cell Mol Gastroenterol Hepatol 1:381-394 e387.
111. Jeanpierre C, Mace G, Parisot M, Moriniere V, Pawtowsky A, Benabou M, Martinovic J, Amiel J, Attie-Bitach T, Delezoide AL, Loget P, Blanchet P, Gaillard D, Gonzales M, Carpentier W, Nitschke P, Tores F, Heidet L, Antignac C, Salomon R, Societe Francaise de F (2011) RET and GDNF mutations are rare in fetuses with renal agenesis or other severe kidney development defects. J Med Genet 48:497-504.
112. Skinner MA, Safford SD, Reeves JG, Jackson ME, Freemerman AJ (2008) Renal aplasia in humans is associated with RET mutations. Am J Hum Genet 82:344-351.
113. Lu W, van Eerde AM, Fan X, Quintero-Rivera F, Kulkarni S, Ferguson H, Kim HG, Fan Y, Xi Q, Li QG, Sanlaville D, Andrews W, Sundaresan V, Bi W, Yan J, Giltay JC, Wijmenga C, de Jong TP, Feather SA, Woolf AS, Rao Y, Lupski JR, Eccles MR, Quade BJ, Gusella JF, Morton CC, Maas RL (2007) Disruption of ROBO2 is associated with urinary tract anomalies and confers risk of vesicoureteral reflux. Am J Hum Genet 80:616-632.

114. Blanck C, Kohlhase J, Engels S, Burfeind P, Engel W, Bottani A, Patel MS, Kroes HY, Cobben JM (2000) Three novel SALL1 mutations extend the mutational spectrum in Townes-Brocks syndrome. J Med Genet 37:303-307.
115. Botzenhart EM, Green A, Ilyina H, Konig R, Lowry RB, Lo IF, Shohat M, Burke L, McGaughran J, Chafai R, Pierquin G, Michaelis RC, Whiteford ML, Simola KO, Rosler B, Kohlhase J (2005) SALL1 mutation analysis in Townes-Brocks syndrome: twelve novel mutations and expansion of the phenotype. Hum Mutat 26:282.
116. Botzenhart EM, Bartalini G, Blair E, Brady AF, Elmslie F, Chong KL, Christy K, Torres-Martinez W, Danesino C, Deardorff MA, Fryns JP, Marlin S, Garcia-Minaur S, Hellenbroich Y, Hay BN, Penttinen M, Shashi V, Terhal P, Van Maldergem L, Whiteford ML, Zackai E, Kohlhase J (2007) Townes-Brocks syndrome: twenty novel SALL1 mutations in sporadic and familial cases and refinement of the SALL1 hot spot region. Hum Mutat 28:204-205.
117. Ferraz FG, Nunes L, Ferraz ME, Sousa JP, Santos M, Carvalho C, Maroteaux P (1989) Townes-Brocks syndrome. Report of a case and review of the literature. Ann Genet 32:120-123.
118. Johnson JP, Poskanzer LS, Sherman S (1996) Three-generation family with resemblance to Townes-Brocks syndrome and Goldenhar/oculoauriculovertebral spectrum. Am J Med Genet 61:134-139.
119. Kohlhase J, Taschner PE, Burfeind P, Pasche B, Newman B, Blanck C, Breuning MH, ten Kate LP, Maaswinkel-Mooy P, Mitulla B, Seidel J, Kirkpatrick SJ, Pauli RM, Wargowski DS, Devriendt K, Proesmans W, Gabrielli O, Coppa GV, Wesby-van Swaay E, Trembath RC, Schinzel AA, Reardon W, Seemanova E, Engel W (1999) Molecular analysis of SALL1 mutations in Townes-Brocks syndrome. Am J Hum Genet 64:435-445.
120. Kurnit DM, Steele MW, Pinsky L, Dibbins A (1978) Autosomal dominant transmission of a syndrome of anal, ear, renal, and radial congenital malformations. J Pediatr 93:270-273.
121. Rossmiller DR, Pasic TR (1994) Hearing loss in Townes-Brocks syndrome. Otolaryngol Head Neck Surg 111:175-180.
122. Hoskins BE, Cramer CH, Silvius D, Zou D, Raymond RM, Orten DJ, Kimberling WJ, Smith RJ, Weil D, Petit C, Otto EA, Xu PX, Hildebrandt F (2007) Transcription factor SIX5 is mutated in patients with branchio-oto-renal syndrome. Am J Hum Genet 80:800-804.
123. Berkenstadt M, Lev D, Achiron R, Rosner M, Barkai G (1999) Pulmonary agenesis, microphthalmia, and diaphragmatic defect (PMD): new syndrome or association? Am J Med Genet 86:6-8.
124. Casey J, Kawaguchi R, Morrissey M, Sun H, McGettigan P, Nielsen JE, Conroy J, Regan R, Kenny E, Cormican P, Morris DW, Tormey P, Chroinin MN, Kennedy BN, Lynch S, Green A, Ennis S (2011) First implication of STRA6 mutations in isolated anophthalmia, microphthalmia, and coloboma: a new dimension to the STRA6 phenotype. Hum Mutat 32:1417-1426.
125. Li L, Wei J (2006) A newborn with anophthalmia and pulmonary hypoplasia (the Matthew-Wood syndrome). Am J Med Genet A 140:1564-1566.
126. Pasutto F, Sticht H, Hammersen G, Gillessen-Kaesbach G, Fitzpatrick DR, Nurnberg G, Brasch F, Schirmer-Zimmermann H, Tolmie JL, Chitayat D, Houge G, Fernandez-Martinez L, Keating S, Mortier G, Hennekam RC, von der Wense A, Slavotinek A, Meinecke P, Bitoun P, Becker C, Nurnberg P, Reis A, Rauch A (2007) Mutations in STRA6 cause a broad spectrum of malformations including anophthalmia, congenital heart defects, diaphragmatic hernia, alveolar capillary dysplasia, lung hypoplasia, and mental retardation. Am J Hum Genet 80:550-560.
127. Smith SA, Martin KE, Dodd KL, Young ID (1994) Severe microphthalmia, diaphragmatic hernia and Fallot's tetralogy associated with a chromosome 1;15 translocation. Clin Dysmorphol 3:287-291.
128. Kosfeld A, Kreuzer M, Daniel C, Brand F, Schafer AK, Chadt A, Weiss AC, Riehmer V, Jeanpierre C, Klintschar M, Brasen JH, Amann K, Pape L, Kispert A, Al-Hasani H, Haffner D, Weber RG (2016) Whole-exome sequencing identifies mutations of TBC1D1 encoding a Rab-GTPase-activating

protein in patients with congenital anomalies of the kidneys and urinary tract (CAKUT). Hum Genet 135:69-87.
129. Demirci H, Shields CL, Shields JA (2005) New ophthalmic manifestations of branchio-oculo-facial syndrome. Am J Ophthalmol 139:362-364.
130. Vivante A, Kleppa MJ, Schulz J, Kohl S, Sharma A, Chen J, Shril S, Hwang DY, Weiss AC, Kaminski MM, Shukrun R, Kemper MJ, Lehnhardt A, Beetz R, Sanna-Cherchi S, Verbitsky M, Gharavi AG, Stuart HM, Feather SA, Goodship JA, Goodship TH, Woolf AS, Westra SJ, Doody DP, Bauer SB, Lee RS, Adam RM, Lu W, Reutter HM, Kehinde EO, Mancini EJ, Lifton RP, Tasic V, Lienkamp SS, Juppner H, Kispert A, Hildebrandt F (2015) Mutations in TBX18 Cause Dominant Urinary Tract Malformations via Transcriptional Dysregulation of Ureter Development. Am J Hum Genet 97:291-301.
131. Fujimoto A, Lipson M, Lacro RV, Shinno NW, Boelter WD, Jones KL, Wilson MG (1987) New autosomal dominant branchio-oculo-facial syndrome. Am J Med Genet 27:943-951.
132. Lee WK, Root AW, Fenske N (1982) Bilateral branchial cleft sinuses associated with intrauterine and postnatal growth retardation, premature aging, and unusual facial appearance: a new syndrome with dominant transmission. Am J Med Genet 11:345-352.
133. Lin AE, Gorlin RJ, Lurie IW, Brunner HG, van der Burgt I, Naumchik IV, Rumyantseva NV, Stengel-Rutkowski S, Rosenbaum K, Meinecke P, et al. (1995) Further delineation of the branchio-oculo-facial syndrome. Am J Med Genet 56:42-59.
134. Richardson E, Davison C, Moore AT (1996) Colobomatous microphthalmia with midfacial clefting: part of the spectrum of branchio-oculo-facial syndrome? Ophthalmic Genet 17:59-65.
135. Ta-Shma A, Khan TN, Vivante A, Willer JR, Matak P, Jalas C, Pode-Shakked B, Salem Y, Anikster Y, Hildebrandt F, Katsanis N, Elpeleg O, Davis EE (2017) Mutations in TMEM260 Cause a Pediatric Neurodevelopmental, Cardiac, and Renal Syndrome. Am J Hum Genet 100:666-675.
136. Saisawat P, Kohl S, Hilger AC, Hwang DY, Yung Gee H, Dworschak GC, Tasic V, Pennimpede T, Natarajan S, Sperry E, Matassa DS, Stajic N, Bogdanovic R, de Blaauw I, Marcelis CL, Wijers CH, Bartels E, Schmiedeke E, Schmidt D, Marzheuser S, Grasshoff-Derr S, Holland-Cunz S, Ludwig M, Nothen MM, Draaken M, Brosens E, Heij H, Tibboel D, Herrmann BG, Solomon BD, de Klein A, van Rooij IA, Esposito F, Reutter HM, Hildebrandt F (2014) Whole-exome resequencing reveals recessive mutations in TRAP1 in individuals with CAKUT and VACTERL association. Kidney Int 85:1310-1317.
137. Martin E, Enriquez A, Sparrow DB, Humphreys DT, McInerney-Leo AM, Leo PJ, Duncan EL, Iyer KR, Greasby JA, Ip E, Giannoulatou E, Sheng D, Wohler E, Dimartino C, Amiel J, Capri Y, Lehalle D, Mory A, Wilnai Y, Lebenthal Y, Gharavi AG, Krzemien GG, Miklaszewska M, Steiner RD, Raggio C, Blank R, Baris Feldman H, Milo Rasouly H, Sobreira NLM, Jobling R, Gordon CT, Giampietro PF, Dunwoodie SL, Chapman G (2020) Heterozygous loss of WBP11 function causes multiple congenital defects in humans and mice. Hum Mol Genet 29:3662-3678.
138. Froster UG, Wallner SJ, Reusche E, Schwinger E, Rehder H (1996) VACTERL with hydrocephalus and branchial arch defects: prenatal, clinical, and autopsy findings in two brothers. Am J Med Genet 62:169-172.
139. Wessels MW, Kuchinka B, Heydanus R, Smit BJ, Dooijes D, de Krijger RR, Lequin MH, de Jong EM, Husen M, Willems PJ, Casey B (2010) Polyalanine expansion in the ZIC3 gene leading to X-linked heterotaxy with VACTERL association: a new polyalanine disorder? J Med Genet 47:351-355.
140. Chung B, Shaffer LG, Keating S, Johnson J, Casey B, Chitayat D (2011) From VACTERL-H to heterotaxy: variable expressivity of ZIC3-related disorders. Am J Med Genet A 155A:1123-1128.
141. Connaughton DM, Dai R, Owen DJ, Marquez J, Mann N, Graham-Paquin AL, Nakayama M, Coyaud E, Laurent EMN, St-Germain JR, Blok LS, Vino A, Klambt V, Deutsch K, Wu CW, Kolvenbach CM, Kause F, Ottlewski I, Schneider R, Kitzler TM, Majmundar AJ, Buerger F, Onuchic-Whitford AC, Youying M, Kolb A, Salmanullah D, Chen E, van der Ven AT, Rao J, Ityel H, Seltzsam S, Rieke JM, Chen J, Vivante A, Hwang DY, Kohl S, Dworschak GC, Hermle T, Alders M,

Bartolomaeus T, Bauer SB, Baum MA, Brilstra EH, Challman TD, Zyskind J, Costin CE, Dipple KM, Duijkers FA, Ferguson M, Fitzpatrick DR, Fick R, Glass IA, Hulick PJ, Kline AD, Krey I, Kumar S, Lu W, Marco EJ, Wentzensen IM, Mefford HC, Platzer K, Povolotskaya IS, Savatt JM, Shcherbakova NV, Senguttuvan P, Squire AE, Stein DR, Thiffault I, Voinova VY, Somers MJG, Ferguson MA, Traum AZ, Daouk GH, Daga A, Rodig NM, Terhal PA, van Binsbergen E, Eid LA, Tasic V, Rasouly HM, Lim TY, Ahram DF, Gharavi AG, Reutter HM, Rehm HL, MacArthur DG, Lek M, Laricchia KM, Lifton RP, Xu H, Mane SM, Sanna-Cherchi S, Sharrocks AD, Raught B, Fisher SE, Bouchard M, Khokha MK, Shril S, Hildebrandt F (2020) Mutations of the Transcriptional Corepressor ZMYM2 Cause Syndromic Urinary Tract Malformations. Am J Hum Genet 107:727-742.

142. Bakrania P, Efthymiou M, Klein JC, Salt A, Bunyan DJ, Wyatt A, Ponting CP, Martin A, Williams S, Lindley V, Gilmore J, Restori M, Robson AG, Neveu MM, Holder GE, Collin JR, Robinson DO, Farndon P, Johansen-Berg H, Gerrelli D, Ragge NK (2008) Mutations in BMP4 cause eye, brain, and digit developmental anomalies: overlap between the BMP4 and hedgehog signaling pathways. Am J Hum Genet 82:304-319.

143. Bennett CP, Betts DR, Seller MJ (1991) Deletion 14q (q22q23) associated with anophthalmia, absent pituitary, and other abnormalities. J Med Genet 28:280-281.

144. Weber S, Taylor JC, Winyard P, Baker KF, Sullivan-Brown J, Schild R, Knuppel T, Zurowska AM, Caldas-Alfonso A, Litwin M, Emre S, Ghiggeri GM, Bakkaloglu A, Mehls O, Antignac C, Network E, Schaefer F, Burdine RD (2008) SIX2 and BMP4 mutations associate with anomalous kidney development. J Am Soc Nephrol 19:891-903.

145. Bellini C, Mazzella M, Arioni C, Fondelli MP, Serra G (2002) "Apple-peel" intestinal atresia, ocular anomalies, and microcephaly syndrome: brain magnetic resonance imaging study. Am J Med Genet 110:176-178.

146. Filges I, Bruder E, Brandal K, Meier S, Undlien DE, Waage TR, Hoesli I, Schubach M, de Beer T, Sheng Y, Hoeller S, Schulzke S, Rosby O, Miny P, Tercanli S, Oppedal T, Meyer P, Selmer KK, Stromme P (2016) Stromme Syndrome Is a Ciliary Disorder Caused by Mutations in CENPF. Hum Mutat 37:711.

147. Keegan CE, Vilain E, Mohammed M, Lehoczky J, Dobyns WB, Archer SM, Innis JW (2004) Microcephaly, jejunal atresia, aberrant right bronchus, ocular anomalies, and XY sex reversal. Am J Med Genet A 125A:293-298.

148. Stromme P, Dahl E, Flage T, Stene-Johansen H (1993) Apple peel intestinal atresia in siblings with ocular anomalies and microcephaly. Clin Genet 44:208-210.

149. van Bever Y, van Hest L, Wolfs R, Tibboel D, van den Hoonaard TL, Gischler SJ (2008) Exclusion of a PAX6, FOXC1, PITX2, and MYCN mutation in another patient with apple peel intestinal atresia, ocular anomalies and microcephaly and review of the literature. Am J Med Genet A 146A:500-504.

150. Waters AM, Asfahani R, Carroll P, Bicknell L, Lescai F, Bright A, Chanudet E, Brooks A, Christou-Savina S, Osman G, Walsh P, Bacchelli C, Chapgier A, Vernay B, Bader DM, Deshpande C, M OS, Ocaka L, Stanescu H, Stewart HS, Hildebrandt F, Otto E, Johnson CA, Szymanska K, Katsanis N, Davis E, Kleta R, Hubank M, Doxsey S, Jackson A, Stupka E, Winey M, Beales PL (2015) The kinetochore protein, CENPF, is mutated in human ciliopathy and microcephaly phenotypes. J Med Genet 52:147-156.

151. Shaheen R, Szymanska K, Basu B, Patel N, Ewida N, Faqeih E, Al Hashem A, Derar N, Alsharif H, Aldahmesh MA, Alazami AM, Hashem M, Ibrahim N, Abdulwahab FM, Sonbul R, Alkuraya H, Alnemer M, Al Tala S, Al-Husain M, Morsy H, Seidahmed MZ, Meriki N, Al-Owain M, AlShahwan S, Tabarki B, Salih MA, Ciliopathy W, Faquih T, El-Kalioby M, Ueffing M, Boldt K, Logan CV, Parry DA, Al Tassan N, Monies D, Megarbane A, Abouelhoda M, Halees A, Johnson CA, Alkuraya FS (2016) Characterizing the morbid genome of ciliopathies. Genome Biol 17:242.

152. Barak H, Huh SH, Chen S, Jeanpierre C, Martinovic J, Parisot M, Bole-Feysot C, Nitschke P, Salomon R, Antignac C, Ornitz DM, Kopan R (2012) FGF9 and FGF20 maintain the stemness of nephron progenitors in mice and man. Dev Cell 22:1191-1207.
153. Schneeberger PE, von Elsner L, Barker EL, Meinecke P, Marquardt I, Alawi M, Steindl K, Joset P, Rauch A, Zwijnenburg PJG, Weiss MM, Merry CLR, Kutsche K (2020) Bi-allelic Pathogenic Variants in HS2ST1 Cause a Syndrome Characterized by Developmental Delay and Corpus Callosum, Skeletal, and Renal Abnormalities. Am J Hum Genet 107:1044-1061.
154. Aalfs CM, van Schooneveld MJ, van Keulen EM, Hennekam RC (1996) Further delineation of the acro-renal-ocular syndrome. Am J Med Genet 62:276-281.
155. Becker K, Beales PL, Calver DM, Matthijs G, Mohammed SN (2002) Okihiro syndrome and acro-renal-ocular syndrome: clinical overlap, expansion of the phenotype, and absence of PAX2 mutations in two new families. J Med Genet 39:68-71.
156. Ferrell RL, Jones B, Lucas RV, Jr. (1966) Simultaneous occurrence of the Holt-Oram and the Duane syndromes. J Pediatr 69:630-634.
157. Halal F, Homsy M, Perreault G (1984) Acro-renal-ocular syndrome: autosomal dominant thumb hypoplasia, renal ectopia, and eye defect. Am J Med Genet 17:753-762.
158. Hwang DY, Kohl S, Fan X, Vivante A, Chan S, Dworschak GC, Schulz J, van Eerde AM, Hilger AC, Gee HY, Pennimpede T, Herrmann BG, van de Hoek G, Renkema KY, Schell C, Huber TB, Reutter HM, Soliman NA, Stajic N, Bogdanovic R, Kehinde EO, Lifton RP, Tasic V, Lu W, Hildebrandt F (2015) Mutations of the SLIT2-ROBO2 pathway genes SLIT2 and SRGAP1 confer risk for congenital anomalies of the kidney and urinary tract. Hum Genet 134:905-916.
159. Bain MD, Winter RM, Burn J (1986) Robinow syndrome without mesomelic 'brachymelia': a report of five cases. J Med Genet 23:350-354.
160. Roifman M, Marcelis CL, Paton T, Marshall C, Silver R, Lohr JL, Yntema HG, Venselaar H, Kayserili H, van Bon B, Seaward G, Consortium FC, Brunner HG, Chitayat D (2015) De novo WNT5A-associated autosomal dominant Robinow syndrome suggests specificity of genotype and phenotype. Clin Genet 87:34-41.
161. Turken A, Balci S, Senocak ME, Hicsonmez A (1996) A large inguinal hernia with undescended testes and micropenis in Robinow syndrome. Clin Dysmorphol 5:175-178.
